# Effect of Anti-inflammatory Treatment with AMD3100 and CX_3_CR1 Deficiency on GABA_A_ Receptor Subunit and Expression of Glutamate Decarboxylase Isoforms After Stroke

**DOI:** 10.1007/s12035-021-02510-x

**Published:** 2021-08-20

**Authors:** Georgios Michalettos, Helene L. Walter, Ana Rita Pombo Antunes, Tadeusz Wieloch, Daniela Talhada, Karsten Ruscher

**Affiliations:** 1 Laboratory for Experimental Brain Research, Division of Neurosurgery, Department of Clinical Sciences, Wallenberg Neuroscience Center, Lund University, BMC A13, S-22184 Lund, Sweden; 2 Department of Neurology, Faculty of Medicine and University Hospital Cologne, University of Cologne, Cologne, Germany; 3 LUBIN Lab - Lunds Laboratorium För Neurokirurgisk Hjärnskadeforskning, Division of Neurosurgery, Department of Clinical Sciences, Lund University, Lund, Sweden

**Keywords:** Stroke recovery, Neuronal plasticity, GABA, GABA_A_, Inhibition, CX3CR1, CXCR4, Glutamate decarboxylase, AMD3100

## Abstract

Following stroke, attenuation of detrimental inflammatory pathways might be a promising strategy to improve long-term outcome. In particular, cascades driven by pro-inflammatory chemokines interact with neurotransmitter systems such as the GABAergic system. This crosstalk might be of relevance for mechanisms of neuronal plasticity, however, detailed studies are lacking. The purpose of this study was to determine if treatment with 1,1′-[1,4-phenylenebis(methylene)]bis[1,4,8,11-tetraazacyclotetradecane] (AMD3100), an antagonist to the C-X-C chemokine receptor type 4 (CXCR4) and partial allosteric agonist to CXCR7 (AMD3100) alone or in combination with C-X3-C chemokine receptor type 1 (CX3CR1) deficiency, affect the expression of GABA_A_ subunits and glutamate decarboxylase (GAD) isoforms. Heterozygous, CX3CR1-deficient mice and wild-type littermates were subjected to photothrombosis (PT). Treatment with AMD3100 (0.5 mg/kg twice daily i.p.) was administered starting from day 2 after induction of PT until day 14 after the insult. At this time point, GABA_A_ receptor subunits (α3, β3, δ), GAD65 and GAD67, and CXCR4 were analyzed from the peri-infarct tissue and homotypic brain regions of the contralateral hemisphere by quantitative real-time PCR and Western Blot. Fourteen days after PT, CX3CR1 deficiency resulted in a significant decrease of the three GABA_A_ receptor subunits in both the lesioned and the contralateral hemisphere compared to sham-operated mice. Treatment with AMD3100 promoted the down-regulation of GABA_A_ subunits and GAD67 in the ipsilateral peri-infarct area, while the β3 subunit and the GAD isoforms were up-regulated in homotypic regions of the contralateral cortex. Changes in GABA_A_ receptor subunits and GABA synthesis suggest that the CXCR4/7 and CX3CR1 signaling pathways are involved in the regulation of GABAergic neurotransmission in the post-ischemic brain.

**Supplementary Information:**

The online version contains supplementary material available at 10.1007/s12035-021-02510-x.

## Background

Stroke remains the leading cause of disability worldwide [1]. Neurological deficits are the result of lost neuronal function and network integrity due to acute tissue demise but also are perpetuated by delayed neuronal dysfunction and delayed neuronal cell death. Neuronal damage and tissue demise in many cases cannot be avoided [2, 3] due to an exaggerated inflammatory response, even though patients have been treated with thrombolytics in the acute phase after stroke onset [4]. Indeed, inflammatory cascades, an increased astroglial and oligodendrocyte reactivity may dampen potential regenerative mechanisms to enhance plasticity mechanisms such as neural circuit remodeling [5, 6]. Hence, the brain recruits repair mechanisms to induce plastic changes such as axonal growth, synaptic remodeling, re-myelination, cortical reorganization, and re-wiring of neural networks to healthy intact tissue [7].

GABA_A_ receptors are involved in neuronal plasticity mechanisms during the recovery phase after stroke. These pentameric ion channels consist of various combinations of 19 different subunits encoded by distinct genes, thus representing high heterogeneity in their functional assembly [8, 9]. Immunohistochemical and autoradiographic studies indicated an overall reduction of functional subunits in both the lesioned and intact hemisphere and a divergent role of the synaptic variant α3 subunit [10, 11]. Following photothrombosis, electrophysiological studies coupled with knockout experiments on the neuronal excitability in the peri-infarct area have demonstrated increased tonic inhibition mediated partially by α5- and δ-containing receptors. Pharmacological antagonism on these receptors resulted in improved outcome [12]. On the other hand, a down-regulation of α4 and δ subunits in cortical layers 2 and 3 in the peri-infarct area after transient middle cerebral artery occlusion (tMCAO) resulted in decreased tonic inhibition. This was associated with improved motor performance but increased susceptibility to epileptic seizures [13].

An increased synaptic inhibition is observed specifically in cortical layer 5 through up-regulation of α1-containing receptors, pharmacological agonism of which improved outcome after stroke [14]. Importantly, changes in the amount of functional α1-subunits do not necessarily correlate with respective mRNA level [15]. The β3 subunits might also be responsive to ischemia [16]. In addition to its eminent role in the regulation of plasticity mechanisms, experimental evidence emerged revealing γ-aminobutyric acid (GABA) signaling as a potential “cross-talker” between inflammatory and plasticity processes [17–19].

Modulation of chemokine pathways has emerged as a promising field promoting recovery after stroke. Regarding the CXCL12/CXCR4/CXCR7 pathway, we have shown that administration of AMD3100, an antagonist of CXCR4 receptor and a partial or allosteric agonist of CXCR7, improved functional outcome in rats subjected to tMCAO [20]. Similar effects have been confirmed in mice subjected to PT [21]. Effects were attributed to suppressed immune and microglial response in the ischemic territory [20, 21]. In addition, the CX3CL1/CX3CR1 chemokine pathway is involved in stroke pathology. Several studies have demonstrated that knockout mice, both in the *cx3cr1* and the *cx3cl1* locus, exhibit decreased brain injury and mortality rates [22–24]. Exogenous administration of CX3CL1 to wild-type mice reduced the ischemic damage following MCAO. In contrast, administration of the ligand to *cx3cl1*^*−/−*^ mice increased the infarct size after the insult [22]. We have shown that CX3CR1 deficiency alters the morphology of microglia after tMCAO, ultimately affecting the microglial response but does not affect infarct size after tMCAO [25].

It is important to understand the mechanisms involved in enhancing plasticity in the post-stroke brain that promote functional outcome after stroke [5]. In this regard, the interplay between chemokine pathways and the GABAergic neurotransmission system has not been addressed. Here, we have investigated the effects of AMD3100 treatment and CX3CR1 deficiency on GABA_A_ subunits in the post-ischemic brain with emphasis on the cortical regions ipsilateral and contralateral to the lesioned hemisphere. We chose to investigate the expression of the α3 subunit, a synaptic variant which has been demonstrated to be relevant to stroke studies regarding its expression in cortical regions, homotopic to the infarct area [10]. In addition, we investigated the potential regulation of the δ subunit as a representative variant of tonic inhibition [8, 12] and the β3 subunit as a representative variant of overall receptor assembly and inhibitory efficacy [26]. In addition, the role of GAD67 and GAD65, enzyme isoforms responsible for converting glutamate to GABA, were investigated as key elements of the GABAergic neurotransmission [27, 28].

## Methods

### Experimental Design

Studies are a continuation of previous investigations showing beneficial effects of AMD3100 following experimental stroke induced by photothrombosis (PT) without affecting infarct size [21]. All animal experiments were approved by the Malmö/Lund ethical committee. Animals were housed in a controlled environment with a 12:12 h light cycle beginning at 07:00 with room temperature maintained at 22 °C and with ad libitum access to food and water.

**Fig. 1:**
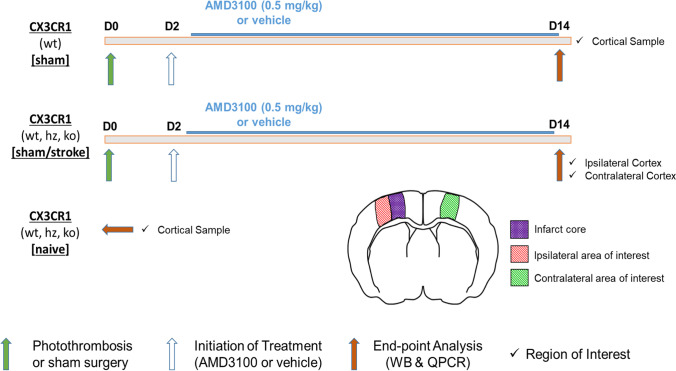
Experimental design. Endpoint analysis included quantification of protein levels by Western Blot and quantification of transcript levels by quantitative polymerase chain reaction. Sham-operated wild-type mice were used to study AMD3100 treatment effects in physiological conditions. Naïve animals did not undergo surgery

As shown in Fig. 1, wild-type [referred to as (wt) mice], CX3CR1 heterozygous [referred to as (hz) mice], and CX3CR1-deficient [referred to as (ko) mice] mice were subjected to permanent focal ischemia induced by photothrombosis (PT). From day 2 to day 14 after induction of stroke/sham surgery, animals were treated with either AMD3100 (every 12 h, 0.5 mg/kg body weight) or saline vehicle (vh). Treatments were administered by intraperitoneal injections twice daily. Naïve animals from all three genotypes were used as controls. On day 14, animals were sacrificed, brains were snap-frozen, stored at – 80 °C, and subsequently, tissue samples from the peri-infarct area and homotypic areas from the non-lesioned hemisphere were used for endpoint analyses (Fig. 1).

### Animals

To study the effect of the CX3CR1 genotype, transgenic C57BL/6 mice, which carried GFP at the gene locus, under the control of the CX3CR1 promoter, were used [29]. Animals were obtained by own breeding. In total, 64 mice were used in which the experimental groups along with their respective treatment consisted of the following: 3 sham/vh mice consisting of 1 wt, 1 Hz and 1 ko mouse, 4 PT/vh-wt mice, 10 PT/vh-hz mice, 5 PT/vh-ko, 5 PT/AMD-wt, 5 PT/AMD-hz, and 5 PT/AMD-ko mice. Fourteen naïve mice were used of which 5 were wt, 4 were hz and 5 were ko on the CX3CR1 locus. In addition, male C57BL/6 mice were used (28 to 35 g, aged 12 to 16 weeks; Charles River, Sulzfeld, Germany) to investigate the effect of AMD3100 treatment. Thirteen animals underwent sham surgery, 7 of which were treated with saline (sham/vh; *n* = 7) and 6 of which were treated with AMD3100 (sham/AMD; *n* = 6).

### Photothrombosis

Photothrombosis was performed as previously described [21]. In brief, animals were initially anesthetized with isoflurane (4 l/min) and placed in a stereotactic frame. During surgery, the animals were spontaneously breathing through a facemask delivering 1.7 L/min of isoflurane in a gas mixture of 30% O_2_ and 70% N_2_O. The surgery started with a sagittal skin incision made on the scalp to expose the skull. Subcutaneous connective tissue was removed and the skullbone was dried. Thereafter, the photosensitive dye Rose Bengal (concentration 10 mg/ml) was injected intraperitoneally. Five minutes after injection, cold light was applied through a round aperture with a diameter of 2.5 mm through the intact skull at the following coordinates (related to bregma): anterior–posterior 1.5 mm and medio-lateral 1 mm. Illumination was carried out for 20 min at an intensity of 3050 K/4D using a Schott cold light source (Schott KL 1500lcd, Mainz, Germany). Following illumination, the scalp incision was sutured, and the mice were transferred to their home cages. Animals subjected to sham surgery were injected intraperitoneally with saline solution. During surgery, the body temperature was monitored through a rectal thermistor probe connected to a heating pad maintaining body temperature between 36.3 and 37.2 °C (Linton Instrumentation, Norfolk, UK). The breathing of the animal was also monitored to adjust anesthesia.

### Genotyping

Genomic DNA was extracted from ear punches. Extraction and PCR were performed using the KAPA Mouse Genotyping Kit (Wilmington, MA, USA) according to the manufacturer’s protocol. Primers CX3CR1_3945 [TTCACGTTCGGTCTGGTGGG], CX3CR1_39456 [GGTTCCTAGTGGAGCTAGGG], and CX3CR1_3947 [GATCACTCTCGGCATGGACG] were used in a final concentration of 500 nΜ. Amplification was carried out in a MJ Mini Personal Thermal Cycler (BioRad, Hercules, USA). Amplified fragments were separated by agarose gel electrophoresis, and results were visualized using a ChemiDoc™ MP system (BioRad).

### Western blot

Tissue samples from snap-frozen brains (isopentane – 70 °C) were microdissected from the peri-infarct area/ipsilateral cortex and the homotypic region of the contralateral hemisphere. Samples were homogenized in lysis buffer (20 mM Tris pH 7.5, 150 mM NaCl, 1 mM EDTA, 1 mM EGTA, 1% Triton X-100, 1 mM β-glycerolphosphate, 1 mM sodium orthovanadate (Na_3_VO_4_), 1 mM phenylmethylsulfonyl fluoride (PMSF), and cOmplete™ Protease Inhibitor Cocktail (Sigma-Aldrich) by sonication and centrifuged at 14.000 RPM for 20 min at 4 °C. The supernatant was collected and stored at – 80 °C.

As indicated in respective figure legends, twenty, thirty, or sixty micrograms of proteins were separated in Mini-Protean® TGX™ and Mini-Protean® TGX Stain-Free™ Gels (Bio-Rad, Hercules, USA) and casted gels. Thereafter, proteins were electro-blotted using a Trans-blot® Turbo™ (Bio-Rad, Hercules, USA) system. After blocking in 5% non-fat dry milk (in TBST), membranes were incubated with primary antibodies including a mouse monoclonal anti-glutamic acid decarboxylase 67 (GAD67) (1:5000; Merck Millipore, Temecula, USA), a rabbit polyclonal anti-GABA_A_ α3 Receptor (1:500; Alomone Labs, Jerusalem, Israel), a rabbit polyclonal anti-GABA_A_ δ Receptor (1:2000, Novus Biologicals, Colorado, USA), a rabbit polyclonal anti-GABA_A_ β3 Receptor (1:2000, Novus Biologicals, Colorado, USA), a rabbit polyclonal anti-CXCR4 (1:1000, Abcam, ab2074, Cambridge, UK), and a rabbit polyclonal anti-GAD65 (1:1000, Novus Biologicals, Colorado, USA) antibody. For the secondary antibody incubation, anti-mouse horse radish peroxidase (HRP) antibody (1:10.000, Sigma-Aldrich, Deisenhofen, Germany) or anti-rabbit HRP antibody (1:12.000; Sigma-Aldrich, Deisenhofen, Germany) were used, respectively. For loading normalization, HRP-conjugated β-actin antibody was used (1:75.000, Sigma-Aldrich, Deisenhofen, Germany). Membranes were exposed on a ChemiDoc™ MP system (BioRad) using a chemiluminescence kit (Merck Millipore, Billerica, MA, USA). Densitometry analysis was conducted using ImageJ software [30].

### Quantitative Real-Time PCR

Extraction of RNA from all tissue brain samples was done with RNeasy® Mini Kit (Qiagen, Hilden, Germany, RefID 74,104) according to the protocol of the manufacturer’s instructions. Dithiothreitol (DTT) was used as a reducing reagent for the isolation of RNA. RNA samples were analyzed for their purity using the NanoDrop (Thermo Scientific, Wilmington, USA). cDNA synthesis was done using iScript cDNA Synthesis Kit (Cat# 1,708,891, Bio-Rad, Hercules, USA) in a MJ Mini Personal Thermal Cycler (Bio-Rad, Hercules, USA) according to the instructions of the manufacturer. Nine-hundred nanograms from each sample were used as template for cDNA synthesis. Samples were diluted to a final concentration of 10 ng/μL.

Quantitative PCR was carried out using the CFX Connect™ Real-Time System (Bio-Rad, Hercules, USA) with SsoAdvanced Universal SYBR® Green Supermix (Cat# 1,725,271, Bio-Rad, Hercules, USA). Sample reaction volume was set to 10 μL. Gene-specific primers were used for the detection of GABA_A_ receptor α3 subunit, δ subunit, β3 subunit, and GAD67 transcripts (online resource). Glyceraldehyde 3-phosphate dehydrogenase (GAPDH) transcript levels were used as the reference gene for normalization. Ten nanograms were used from each sample for every respective reaction.

Transcript levels were amplified by applying the following PCR protocol parameters: 35 cycles consisting of an initial step at 95 °C for 30 s, cycle denaturation at 95 °C for 5 s, and 57 °C (α3, δ, β3, GAPDH), 56 °C (CXCR4), or 58 °C (GAD67) annealing/extension stage for 30 s. External standard curves were used for the estimation of the reaction amplification efficiency using a pool of all the samples and consecutive dilutions of 1, 1/2, 1/5, 1/10, 1/20, and 1/50 of the initial pooled sample. Ct values were plotted against the log_10_ of these dilutions for the calculation of the slope. Concentration of primers specific to GABA_A_ subunits and CXCR4 was set to 200 nM and 300 nM for GAD67 transcripts. Primer concentration for GAPDH reactions was set to 500 nM.

Efficiency of each primer pair was calculated from the slope using the formula [*E* =  − 1 + 10^−1/slope^] [31]. All reaction efficiencies varied between 99 and 107%. Ct values were converted to fluorescence values based on the exponential amplification formula [*Rn* = *R*_0_ (1 + *E*) ^*n*], where *E* is the efficiency of the reaction, *n* is the Ct value, *Rn* is the threshold fluorescence value from which the Ct value is derived, and *R*_0_ is the target initial fluorescence value of the sample, corresponding to cDNA levels [32]. Gene expression data was calculated as the ratio of target gene transcript levels to GAPDH transcript levels and are expressed as means ± standard error of the mean (S.E.M.).

### Statistical Analysis

Statistical analysis was performed using GraphPad Prism 8. Expression levels of genes within each hemisphere were evaluated by one-way analysis of variance (ANOVA) and post hoc multiple comparisons were performed with Tukey’s test (significance level 0.05, confidence interval 0.95). For the comparison of the expression levels between the ipsilateral and the contralateral cortex, two-way analysis of variance (ANOVA) was used along with post hoc Sidak’s multiple comparison test. Western blot results were evaluated either by Students *t* test (significance level 0.05, confidence interval 0.95) or ANOVA and post hoc Tukey’s test. All data are expressed as the mean ± S.E.M.

## Results

### *Effect of CX3CR1 Genotype on GABA*_*A*_* Subunits and GAD67 and GAD65 Expression*

**Fig. 2:**
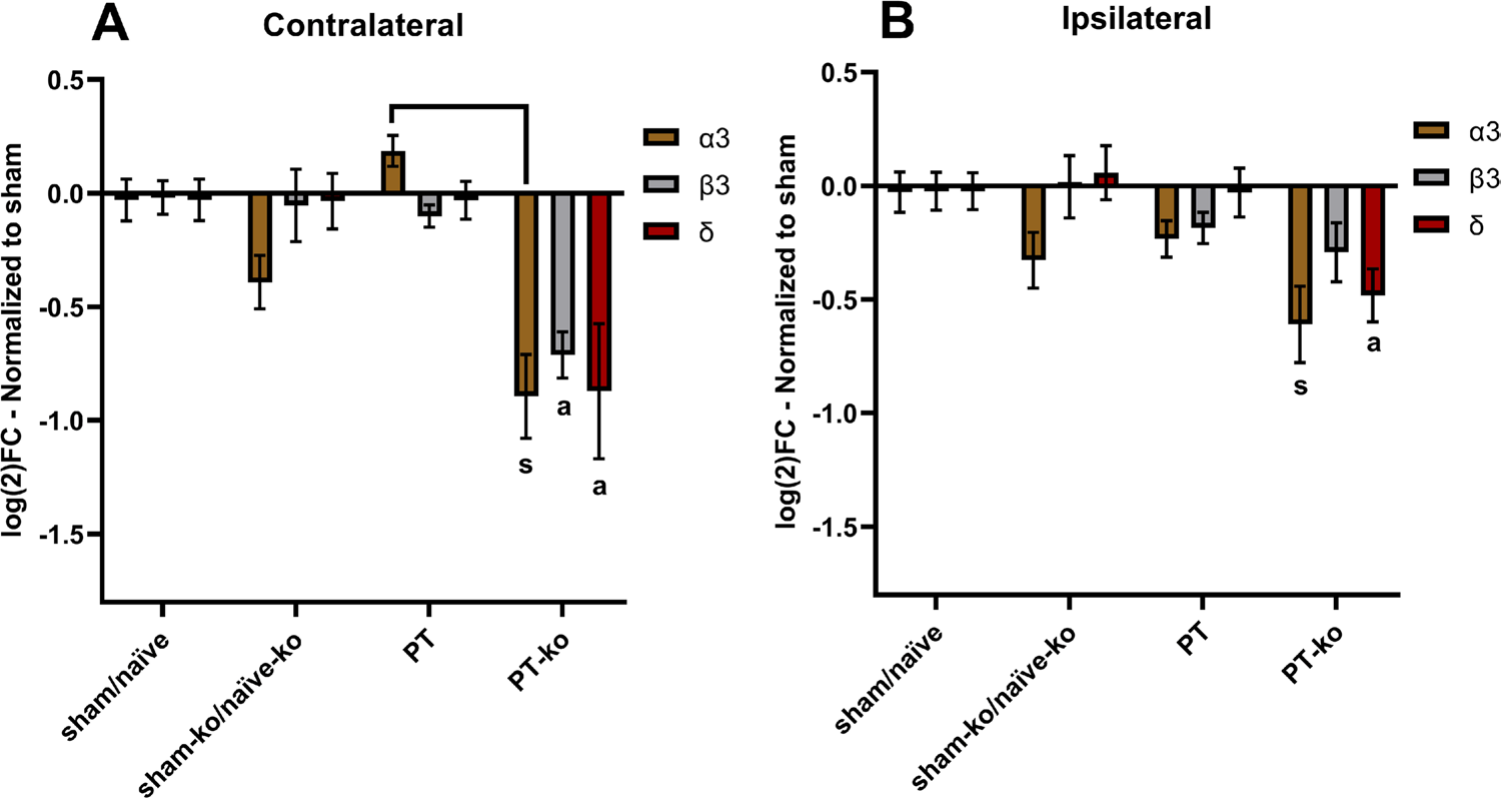
Expression of GABA_A_ subunits in CX3CR1 deficient mice. **A** Effect of receptor deficiency on mRNA levels of the contralateral cortex and **B** ipsilateral cortex. All data depicted are normalized to expression levels of sham/naïve animals and shown as a log_2_ fold-change. All animals corresponding to the experimental groups received i.p. saline injections except naïve animals. Bars represent the mean ± S.E.M. [contralateral: sham/naïve *n* = 18 (8 sham-wt, 1 sham-hz, 5 naïve-wt, 4 naïve-hz), sham-ko/naïve-ko *n* = 6 (1 sham-ko, 5 naïve-ko), PT *n* = 13 (4 wt, 9 Hz), PT-ko *n* = 5; ipsilateral: sham/naïve *n* = 18 (8 sham-wt, 1 sham-hz, 5 naïve-wt, 4 naïve-hz), sham-ko/naïve-ko *n* = 6 (1 sham-ko, 5 naïve-ko), PT *n* = 14 (4 wt, 10 Hz), PT-ko *n* = 5]. CX3CR1-deficient mice subjected to PT exhibited significantly lower mRNA levels in all GABA_A_ subunits compared to sham/naïve levels [s: *(p* < *0.01)*]. Subunits labelled with [a] exhibit significant difference compared to all groups. Additional brackets indicate significant differences between respective groups. [contralateral α3: PT-ko vs PT *(p* < *0.0001)*; contralateral β3: PT-ko vs sham-ko/naïve-ko & PT-ko vs PT *(p* < *0.005)*; contralateral δ: PT-ko vs PT *(p* = *0.007)* & PT-ko vs sham-ko/naïve-ko *(p* < *0.022*); ipsilateral α3: PT-ko vs sham/naïve *(p* = *0.009)*; ipsilateral δ: PT-ko vs PT *(p* = *0.001)* & PT-ko vs sham-ko/naïve-ko *(p* = *0.01)*]

In order to identify alterations in the gene expression of various GABA_A_ subunits in the post-ischemic brain, mRNA levels were quantified in the peri-infarct area, the homotypic region of the contralateral cortex, and respective regions in sham-operated and naïve mice (Fig. 1). *Cx3cr1* knockout mice subjected to PT showed a decrease in the mRNA levels of the GABA_A_ subunits α3, β3, and δ in the ipsi- and contralateral hemisphere compared to sham/naïve mice of both genotypes (Fig. 2). This decrease was more profound on the contralateral non-lesioned cortex with knockout mice subjected to PT exhibiting significantly lower mRNA levels of all three GABA_A_ subunits compared to wt/hz littermates after PT (Fig. 2). In both hemispheres, mRNA levels of the GABA_A_ δ subunit in *cx3cr1* knockout mice subjected to PT were significantly lower compared to all experimental groups (Fig. 2).

Throughout all experimental groups, no difference was observed for the β3 between the peri-infarct area and corresponding regions from sham/naïve mice (Fig. 2). In addition, we did not find differences between wt and hz mice in regard to all three subunits and experimental conditions. Thus, these mice were pooled for the analysis. Finally, no significant differences were found between naïve knockout mice and knockout mice subjected to sham surgery compared to wt/hz littermates (Fig. 2). Together, these experiments support the idea that *cx3cr1* deletion contributes in the regulation of GABA_A_ receptor subunits synthesis in the recovery phase of stroke.

**Fig. 3:**
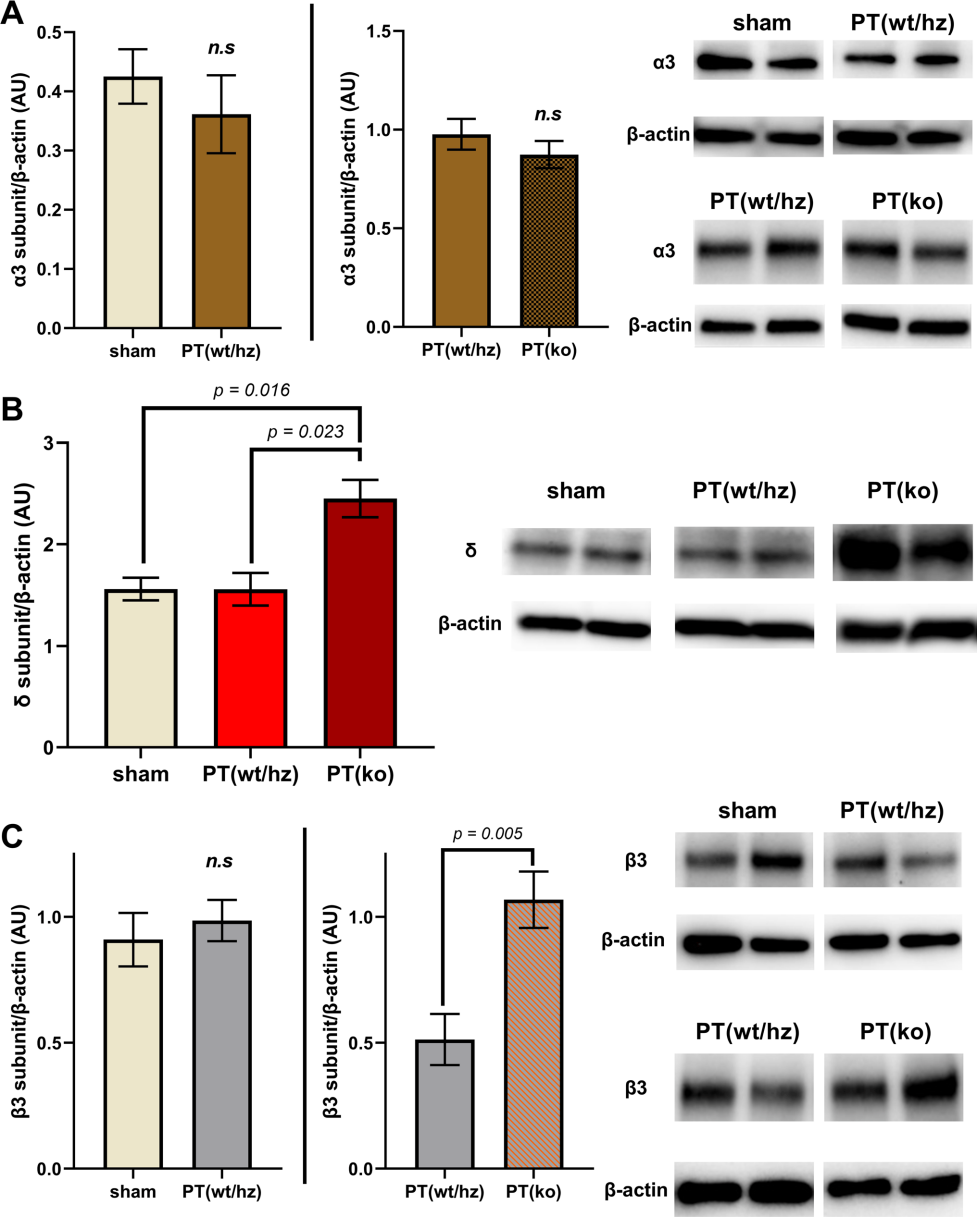
Effect of CX3CR1 deficiency on protein levels of GABA_Α_ subunits on day 14 after stroke. Protein levels of GABA_A_ α3, δ subunits in the contralateral cortex of sham and stroke mice. Specific bands for α3 subunit (55 kDa), δ subunit (52 kDa), b3 subunit (55 kDa), and β-actin were quantified and presented as the percentage ratio of β-actin (AU) as mean ± S.E.M. Protein sample amount used for the α3 subunit was 30 μg, 60 μg for the δ subunit, and 20 μg for the β3 subunit. Representative band images used in each separate graph were taken from the same membrane. Each graph represents a separate Western Blot experiment. All animals corresponding to the experimental groups received i.p. saline injections. [contralateral α3: sham n = 3 (2 wt, 1 Hz) vs PT(wt/hz) n = 4 (2 wt, 2 Hz), PT(wt/hz) n = 6 (4 wt, 2 Hz) vs PT(ko) n = 5. No statistical differences were observed using Student’s test; (sham vs PT(wt/hz) p = 0.50, PT(wt/hz) vs PT(ko), p = 0.35)]. [contralateral δ: sham n = 3 (2 wt, 1 Hz), PT(wt/hz) n = 5 (3 wt, 2 Hz), PT(ko) n = 5. Statistical differences were observed using ANOVA (p = 0.005) and post hoc Tukey’s multiple comparison test; sham vs PT(wt/hz) p > 0.999]. [contralateral β3: sham n = 5 (2 wt, 3 Hz) vs PT(wt/hz) n = 6 (3 wt, 3 Hz), PT(wt/hz) n = 6 (3 wt, 3 Hz) vs PT(ko) n = 5. Statistical differences were evaluated by Student’s test. sham vs PT(wt/hz) p > 0.58]

Transcriptional data prompted us to determine protein levels of the three GABA_A_ subunits in the contralateral cortex of wild-type/heterozygous and *cx3cr1* knockout mice. As shown in Fig. 3, no difference was observed between the experimental groups regarding the synaptic GABA_A_ α3 subunit. In addition, we also did not find differences in the levels of the α3 subunit comparing sham-operated/naïve animals with wt/hz mice subjected to PT in the contralateral cortex. In contrast to the transcriptional data, we observed increased protein levels of the δ and β3 subunit in knockout mice both compared to wt/hz littermates subjected to PT (Fig. 3). No differences were found between naïve/sham-operated mice and mice subjected to PT (Fig. 3). These findings indicate that the CX3CR1 pathway may regulate the transcription of GABA_A_ subunit as well as it may modulate the composition of GABA_A_ receptors on the post-translational level.

Moreover, CX3CR1 deficiency did not affect the transcriptional and posttranscriptional regulation of GAD67 (data not shown); no difference in GAD65 protein levels was found comparing CX3CR1-deficient mice with wt/hz littermates (online resource).

### *Effect of AMD3100 Treatment on GABA*_*A*_* Subunit Expression*

**Fig. 4:**
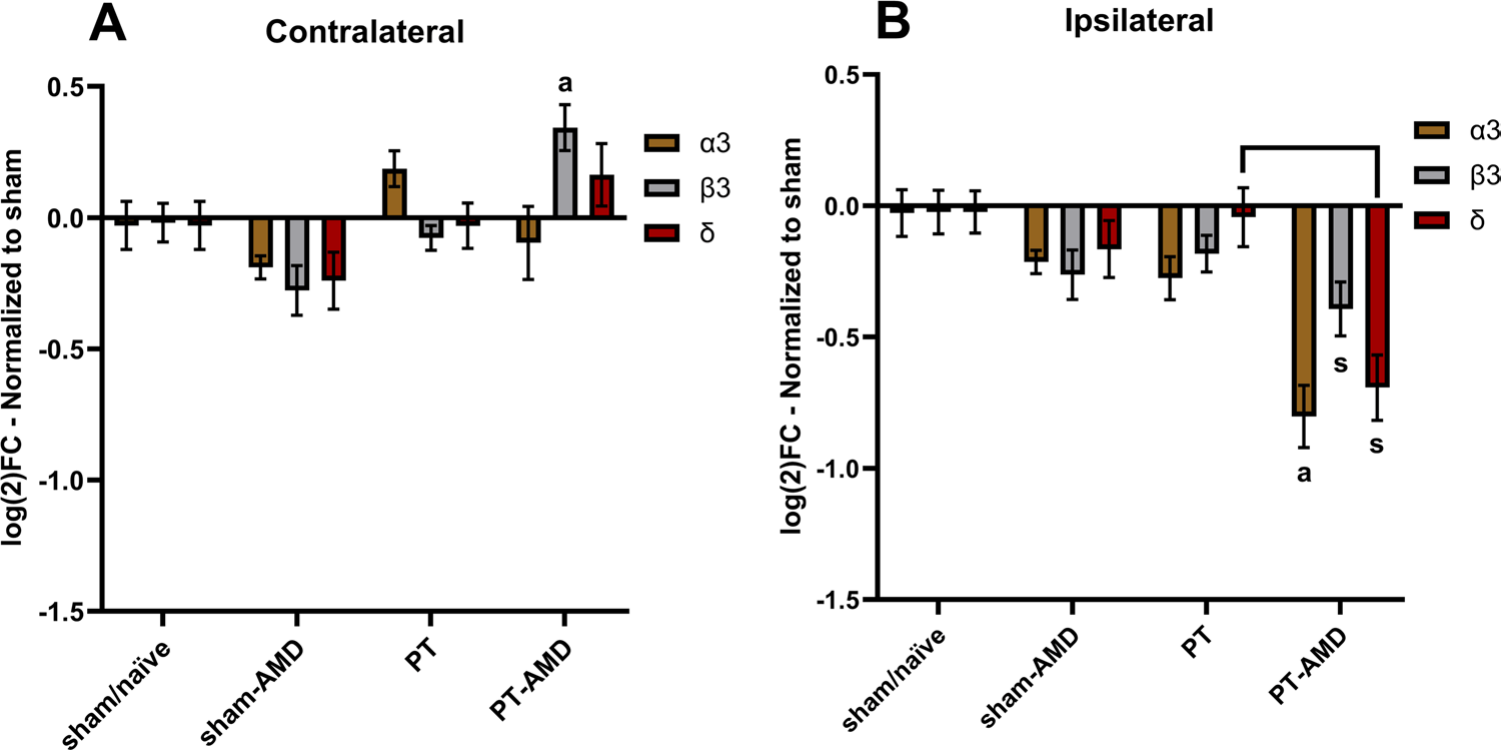
Effect of AMD3100 treatment on the expression of GABA_A_ subunits on day 14 after stroke. mRNA levels of GABA_A_ subunits in the contralateral (**A**) and in the ipsilateral (**B**) cortex. All data are normalized to expression levels of sham/naïve animals and shown as a log_2_ fold-change. Bars represent the mean ± S.E.M. Animals not treated with AMD3100 received i.p. saline injections. [contralateral: sham/naïve *n* = 18 (8 sham-wt, 1 sham-hz, 5 naïve-wt, 4 naïve-hz), sham/AMD *n* = 6 (all wt), PT *n* = 13 (4 wt, 9 Hz), PT/AMD *n* = 10 (5 wt, 5 Hz); ipsilateral: sham/naïve *n* = 18 (8 sham-wt, 1 sham-hz, 5 naïve-wt, 4 naïve-hz), sham/AMD *n* = 6 (all wt), PT *n* = 14 (4 wt, 10 Hz), PT/AMD *n* = 10 (5 wt, 5 Hz)]. Mice subjected to PT and treated with AMD3100 exhibited significantly lower mRNA levels in all GABA_A_ subunits compared to sham/naïve levels in the ipsilateral hemisphere *(p* < *0.05).* Subunits labeled with [a] exhibit significant difference compared to all groups. Additional brackets indicate significant differences between respective groups. [contralateral β3: PT-AMD vs all groups *(p* < *0.005)*, ipsilateral α3: PT-AMD vs sham-AMD *(p* < *0.015)* & PT-AMD vs PT *(p* = *0.004)*, ipsilateral δ: PT-AMD vs PT *(p* = *0.001)*

As previously been reported, the CXCL12/CXCR4 pathway directly affects neuronal function [33]. Here, we evaluated if treatment with the CXCR4 antagonist AMD3100 affected the composition of GABA_A_ receptors. Upon treatment, we found an up-regulation of the β3 subunit in the contralateral cortex (Fig. 4A) and a down-regulation of all three subunits in the peri-infarct area (Fig. 4B). In specific, treated mice subjected to PT exhibited significantly lower transcript levels of the GABA_Α_ α3 and δ subunits in the peri-infarct area, both compared to sham/naïve mice and untreated mice subjected to PT. In addition, these experiments showed no differences in transcript quantities of untreated mice 14 days after PT compared to sham/naïve animals (Fig. 4). Together, these results point towards a down-regulation of GABA_A_ receptors in the ipsilateral and an up-regulation in the contralateral hemisphere after PT upon treatment with AMD3100, respectively.

### Expression of CXCR4 in the Post-Ischemic Brain

**Fig. 5:**
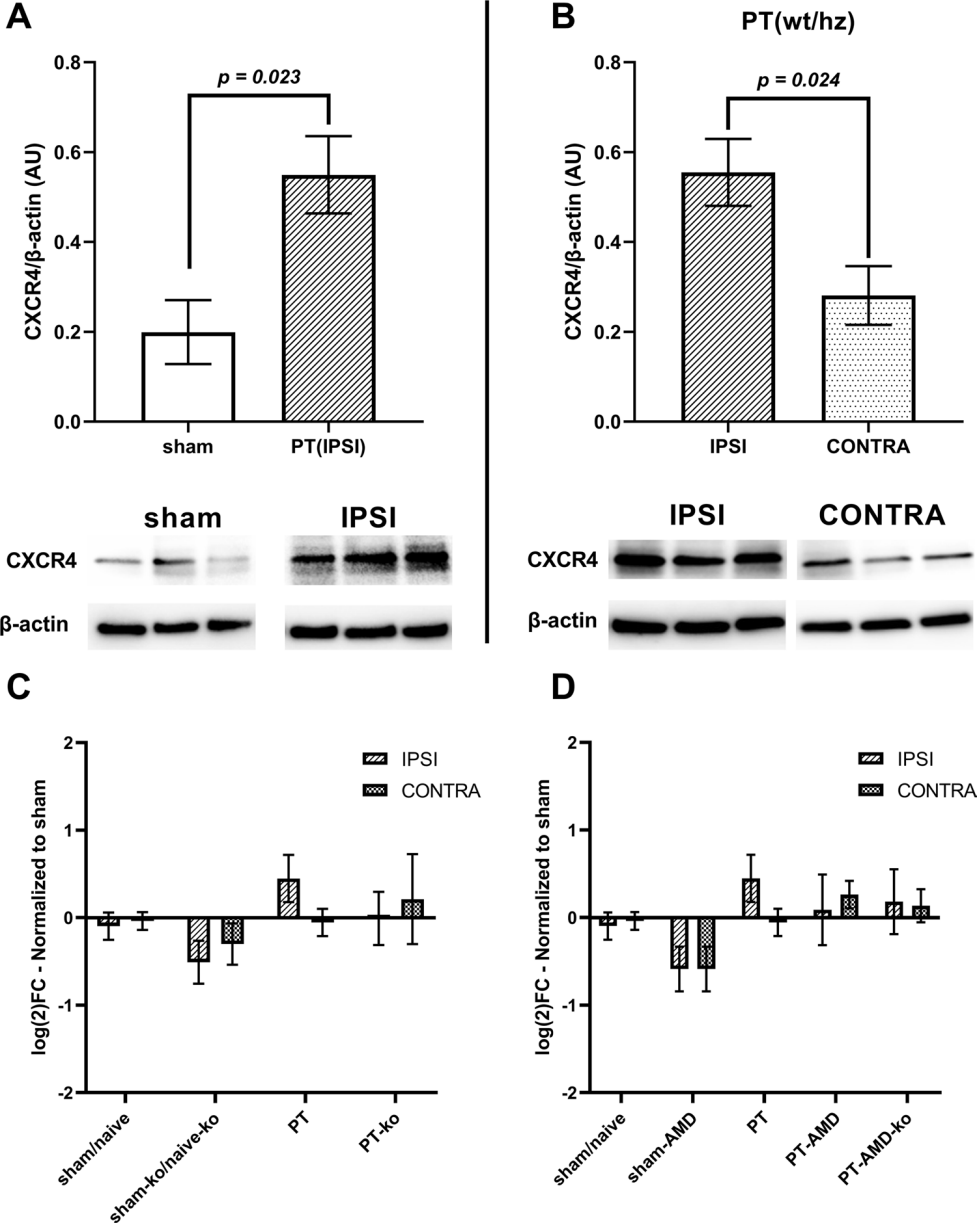
Levels of CXCR4 in the post-ischemic brain. Comparison of protein levels of CXCR4 between the cortex of sham mice and the ipsilateral cortex of stroke mice (**A**) and between the contralateral and the ipsilateral cortex (**B**) after PT. Twenty micrograms of sample protein for used for CXCR4. [sham *n* = 4 (2 wt, 2 Hz) vs PT-IPSI *n* = 7 (4 wt, 3 Hz), PT-IPSI *n* = 6 (4 wt, 2 Hz) vs PT-CONTRA *n* = 5 (2 wt, 3 Hz)]. Effect of CX3CR1 deficiency on transcript levels of CXCR4 (**C**) and effect of AMD3100 treatment on transcript levels of CXCR4 (**D**). Data are normalized to expression levels of sham/naïve animals and shown as a log2 fold-change. Bars represent the mean ± S.E.M. Animals not treated with AMD3100 received i.p. saline injections**.** Naïve animals did not receive any injections. [contralateral: sham/naïve *n* = 11 (1 sham-wt, 1 sham-hz, 5 naïve-wt, 4 naïve-hz), sham-ko/naïve-ko *n* = 6 (1 sham-ko, 5 naïve-ko), sham/AMD *n* = 6 (all wt), PT *n* = 12 (4 wt, 8 Hz), PT-ko *n* = 5, PT-AMD *n* = 5 (all wt), PT-AMD-ko *n* = 5]. [ipsilateral: sham/naïve *n* = 11 (1 sham-wt, 1 sham-hz, 5 naïve-wt, 4 naïve-hz), sham-ko/naïve-ko *n* = 6 (1 sham-ko, 5 naïve-ko), sham/AMD *n* = 6 (all wt), PT *n* = 14 (4 wt, 10 Hz), PT-ko *n* = 5, PT-AMD *n* = 5 (all wt), PT-AMD-ko *n* = 5]. No significant differences were neither observed by post hoc Tukey’s multiple comparisons following One-way ANOVA in respect to each hemisphere nor by Sidak’s multiple comparisons following two-way ANOVA in respect to the two hemispheres

Gene expression and protein levels of CXCR4 were determined between the ipsi- and contralateral hemisphere following PT. As shown in Fig. 5A, we observed increased CXCR4 protein levels in the peri-infarct area of mice subjected to PT, 14 days after stroke, compared to sham-operated mice. In addition, CXCR4 protein levels in the ipsilateral cortex were significantly higher compared to the contralateral intact cortex, in wt/hz mice subjected to PT (Fig. 5B). Expression of CXCR4 in healthy cortical tissue of the non-lesioned hemisphere, even to a lesser extent, confirms the presence of CXCR4 in the contralateral cortex.

As such, we decided to investigate possible alterations of CXCR4 transcript levels across all the experimental groups. No significant differences were observed between any groups, both in regard toCX3CR1 deficiency and AMD3100 treatment (Fig. 5C, D). These experiments confirm the presence of the receptor in cells located in regions contralateral to the site of injury.

### Effect of AMD3100 Treatment on GAD67 and GAD65 Expression

**Fig. 6:**
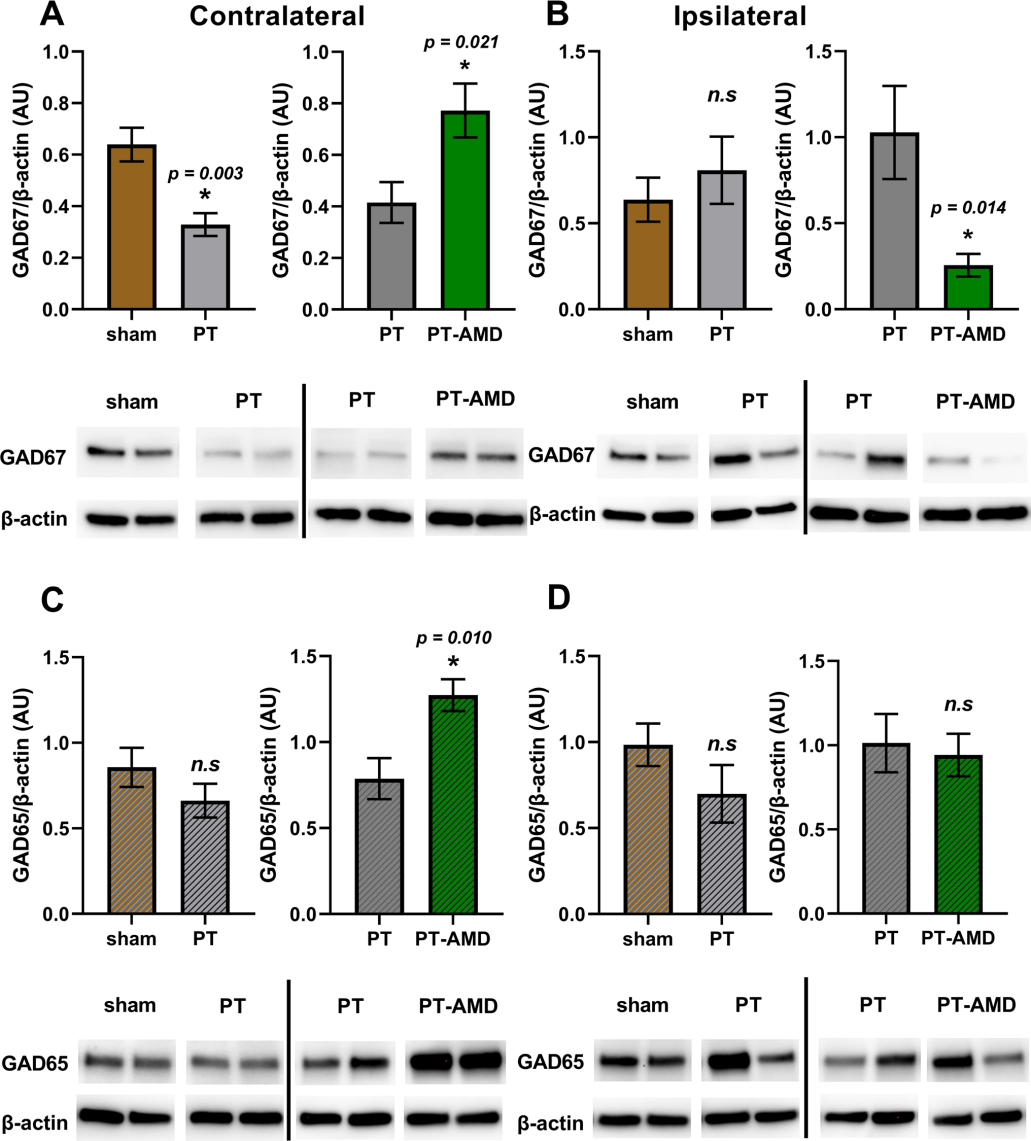
Effect of AMD3100 treatment on levels of GAD67 and GAD65 on day 14 after stroke. Levels of GAD67 and GAD65 in the contralateral (**A, C**) and ipsilateral (**B, D**) cortex of mice subjected to PT and treated with AMD3100 (AMD) i.p injections for 12 days starting on day 2 after PT. Non-treated mice received saline i.p. injections. Specific bands for GAD67 (67 kDa) and GAD65 (65 kDa) and β-actin were quantified and presented as the percentage ratio of β-actin (AU) as mean ± S.E.M. Twenty micrograms of protein were used for both GAD67 and GAD65. Representative band images used in each separate graph were taken from the same membrane. Each graph represents a separate Western Blot experiment. Statistical differences were evaluated by Student’s test. [contralateral GAD67: sham n = 5 (3 wt, 2 Hz), PT n = 6 (3 wt, 3 Hz), PT-AMD n = 5 (4 wt, 1 Hz); ipsilateral GAD67: sham n = 5 (3 wt, 2 Hz), PT n = 6 (4 wt, 2 Hz), PT-AMD n = 5 (3 wt, 2 Hz)]. [contralateral GAD65: sham n = 5 (3 wt, 2 Hz) vs PT n = 6 (3 wt, 3 Hz), PT n = 5 (1 wt, 4 Hz) vs PT-AMD n = 6 (3 wt, 3 Hz); ipsilateral GAD65: sham n = 5 (3 wt, 2 Hz) vs PT n = 6 (4 wt, 2 Hz), PT n = 5 (4 wt, 1 Hz) vs PT-AMD n = 6 (4 wt, 2 Hz)]

Likewise, we observed an opposite effect of AMD3100 on the protein levels of the GABA synthesis regulatory enzyme GAD67 in the peri-infarct area and contralateral cortex (Fig. 6). Western blot experiments were separated in a way to investigate two conditions at a time. In particular, we found similar levels of GAD67 in the ipsilateral peri-infarct area of vehicle treated-mice subjected to PT (0.81 ± 0.2, *n* = 6) compared to sham-operated mice (0.64 ± 0.13, *n* = 5) (Fig. 6B). In contrast, levels were decreased in the contralateral cortex (0.33 ± 0.04, *n* = 6) compared to sham operated mice (0.64 ± 0.07, *n* = 5) (Fig. 5A). Treatment with AMD3100 significantly reduced the levels of GAD67 in the peri-infarct area (0.27 ± 0.07, *n* = 6) compared to vehicle-treated mice (1.03 ± 0.27, *n* = 5) (Fig. 6B). Levels of GAD67 were significantly higher in AMD-treated mice in the contralateral cortex (0.77 ± 0.17, *n* = 5) compared to vehicle-treated animals (0.42 ± 0.08, *n* = 6) after PT (Fig. 6A). Regulation of GAD67 was purely post-transcriptional, since mRNA levels of GAD67 transcripts were similar between the experimental groups (data not shown).

Regarding the protein levels of GAD65, similar levels were found in the ipsilateral peri-infarct area of vehicle treated-mice subjected to PT (0.70 ± 0.17, *n* = 6) compared to sham-operated mice (0.98 ± 0.12, *n* = 5) (Fig. 6D). Similarly, we found no significant differences in levels of GAD65 in the contralateral cortex (0.66 ± 0.10, *n* = 6) compared to sham-operated mice (0.86 ± 0.11, *n* = 5) (Fig. 6C). Contrary to the effect of AMD3100 on GAD67 levels in the peri-infarct area, treatment did not change GAD65 levels in AMD-treated mice (0.94 ± 0.13, *n* = 6) compared to vehicle-treated animals (1.01 ± 0.17, *n* = 5) subjected to PT (Fig. 6D). However, in parallel to the up-regulation of GAD67 in the contralateral cortex due to treatment with AMD3100, we also observed significantly higher levels of GAD65 in the contralateral cortex of AMD-treated mice (1.27 ± 0.09, *n* = 6) compared to vehicle-treated mice (0.79 ± 0.12, *n* = 5) after PT (Fig. 6C).

**Fig. 7:**
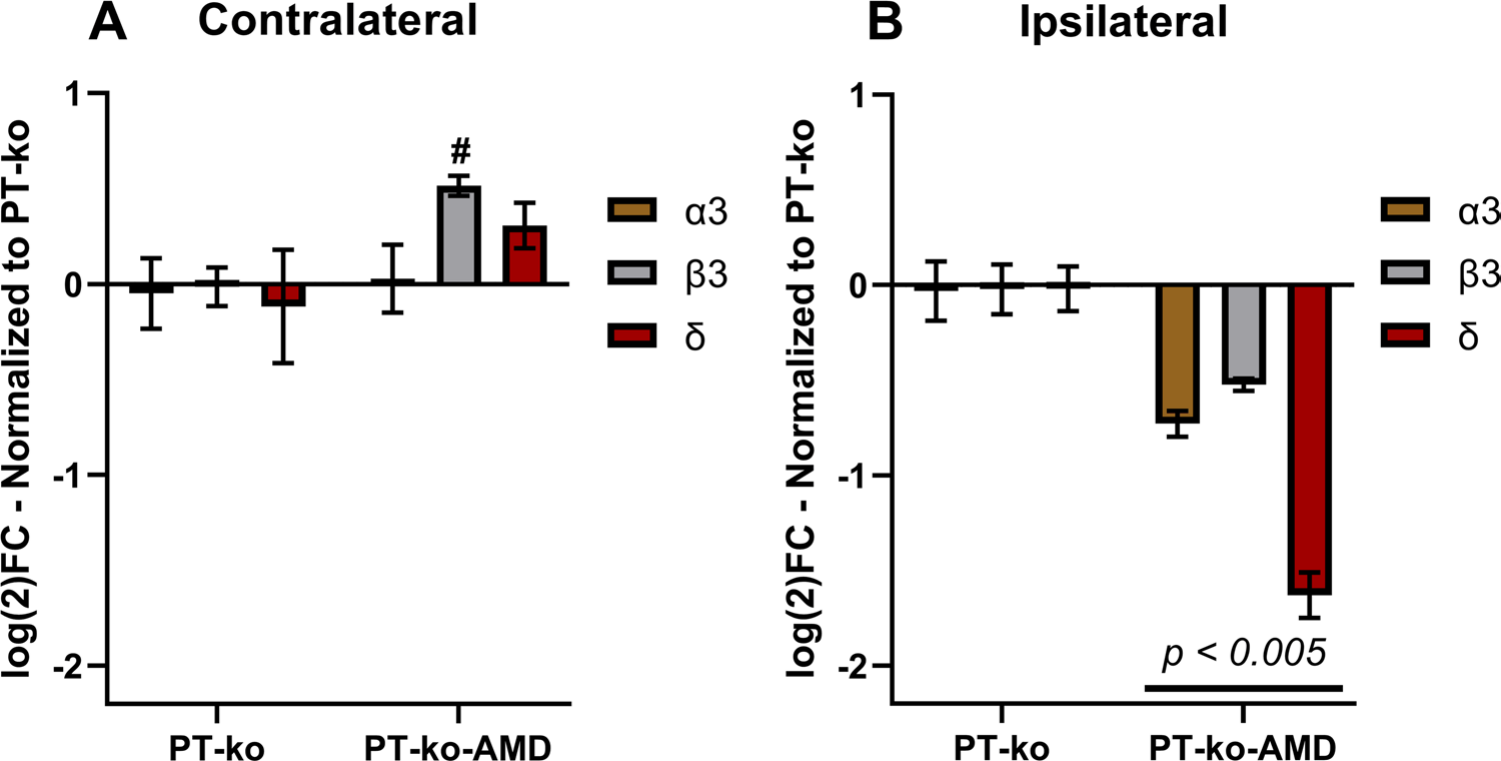
Effect of AMD3100 treatment on post-ischemic CX3CR1-deficient mouse brain. mRNA levels of GABA_A_ subunits in the contralateral (**A**) and ipsilateral (**B**) cortex from mice deficient for CX3CR1 and subjected to PT. All data are normalized to expression levels of PT-ko mice and shown as a log_2_ fold-change. Deficient mice subjected to PT and treated with AMD3100 exhibited significantly lower mRNA levels in all GABA_A_ subunits in the ipsilateral cortex compared to untreated (saline) deficient mice *(p* < *0.005).* [contralateral: PT-ko *n* = 5, PT-AMD-ko *n* = 5; ipsilateral: PT-ko *n* = 5, PT-AMD-ko *n* = 5]. Additional symbols indicate significant differences between respective groups. [#: PT-ko vs PT-AMD-ko *(p* = *0.001)*]. Statistical differences were evaluated by Student’s test

In order to investigate the effect of the treatment together with CX3CR1 deficiency in the post-ischemic brain, expression of gene transcripts for the α3, β3, and δ subunits were analyzed in the ipsilateral peri-infarct area and the contralateral cortex (Fig. 7). Compared to vehicle-treated mice, the expression of all three subunits was significantly reduced in the peri-infarct area following treatment with AMD3100. The opposite effect was found in the contralateral hemisphere showing a significant higher expression of the β3 subunit. The other two subunits remained unchanged compared to levels found in vehicle treated *cx3cr1* knockout mice. These results point towards an additive effect of AMD3100 treatment on the down-regulation of the three GABA_A_ subunits in the ischemic hemisphere of *cx3cr1* knockout mice.

A more comprehensive view on differences in GABA_A_ receptor subunit expression in the lesioned ipsilateral and the non-lesioned contralateral hemisphere after PT is shown in the online resource. Based on this compilation, a significant difference of mRNA levels of the synaptic α3 subunit between the contralateral and ipsilateral cortex on day 14 after PT and in respective regions of mice subjected to PT and treated with AMD3100 becomes evident (Online resource 1). Moreover, compiled data again show the general down-regulation of GABA_A_ receptor subunit transcripts in *cx3cr1* knockout mice and additional down-regulation by treatment with AMD3100. This additive effect of the two experimental conditions seems to be especially high on the down-regulation of GABA_A_ δ subunit transcripts in the peri-infarct area (online resource). Treatment also had an opposite effect of receptor subunits expression in the ipsi- and contralateral hemisphere with higher expression levels in the contralateral cortex.

## Discussion

The present study has been conducted based on previous studies providing evidence for an interaction between the CXCL12/CXCR4/7 and the fractalkine/CX3CR1 chemokine pathways with GABAergic neurotransmission. Here, we investigated if CX3CR1 deficiency and treatment with AMD3100, an antagonist on the CXCR4 and partial allosteric agonist on the CXCR7 affects the expression of the GABA_A_ receptor subunits α3, β3, and δ and the synthesis of GABA by regulation of the glutamic acid decarboxylase isoforms 67 and 65. Our results indicate that CX3CR1 deficiency alters the expression of GABA_A_ subunits in the post-ischemic brain. In addition, treatment with AMD3100 decreased the expression of receptor subunits in the ipsilateral peri-infarct area, while an increased expression was found in the non-lesioned contralateral hemisphere.

### *GABA*_*A*_* Receptor Composition and GAD67/65 Regulation After PT: Effects of AMD3100 Treatment*

Scientific and clinical evidence point towards an excitatory/inhibitory (E/I) imbalance between the lesioned and contralateral hemisphere in the post-ischemic brain [34, 35]. It is characterized by an increased activity (excitation, E) in the contralateral hemisphere exerting inhibition towards the lesioned hemisphere. This model of interhemispheric imbalance is believed to impair neuronal plasticity mechanisms required to remodel neuronal connections and circuits [36]. Structural units to maintain inhibition are respective synapses containing GABA_A_ receptors and GAD isoforms as key mediators of both fast-paced and long-term inhibition. Receptor subunits are also characterized by several modes of regulation, including transcriptional control, post-transcriptional, and post-translational modifications [37–39]. As such, GABA_A_ receptor assembly dynamics can be modulated on multiple levels.

Previous studies have associated either pharmacologically induced reduction of tonic inhibition or spontaneously reduced tonic inhibition in the peri-infarct area with improved functional outcome [12, 13]. Here, we found that AMD3100 reduces the expression of the δ-subunit, involved in tonic inhibition, in the ipsilateral peri-infarct area. This effect could complement attenuated inflammatory cascades achieved by the treatment, ultimately resulting in an improved outcome [20].

The β3 subunit has been proposed to possess a vital role in mediating inhibitory transmission through its assembly with the majority of functional receptors, synaptic or extra-synaptic [26]. For this reason, changes in the levels of the β3 subunit could reflect changes in the overall quantity of functional receptors or total inhibitory efficiency. Furthermore, different expression levels of the β3 subunit between the two hemispheres could indicate transcriptional control as a downstream effect of CXCR4 signaling. In addition, we observed an asymmetry in the expression of the synaptic α3 subunit of vehicle-treated mice subjected to PT (online resource). Differential expression in the ipsi- and contralateral hemisphere might be interpreted as an innate attempt to lower the inhibitory tonus in the peri-infarct area and to promote an increased inhibition in the contralateral cortex.

Following stroke, an accumulation of CXCR4 positive cells, including neurons, is observed in the peri-infarct area accompanied with increased levels of CXCL12, the endogenous ligand of the CXCR4 [20, 40, 41]. In contrast, these changes are not found in homotypic regions of the contralateral cortex. Similar to the results of this study, the higher amount of CXCR4 receptors present in the peri-infarct area, 2 weeks after PT, points towards an accumulation of CXCR4 positive cells. Therefore, differential patterns of CXCR4-expressing cells along with different levels of CXCL12 throughout the ischemic brain might explain different compositions of GABA_A_ receptors in different regions and could also explain the different effect of the treatment on the expression profile of GABA_A_ receptor subunits.

The presence of CXCR4^+^ cells in the ipsilateral non-lesioned cortex, both during the acute and subacute phase [21, 40, 41], indicates the existence of viable targets for AMD3100 during the first weeks after an ischemic insult. Recent studies have extensively delineated the composition of immune CXCR4^+^ cells that accumulate in the area in and adjacent to the infarct core during the acute phase and that CXCR4 can be used as marker to distinguish microglia from infiltrating monocytes in the ischemic territory [42]. Specifically, it was shown that monocytes greatly populate the peri-infarct area at day 3 after PT. The majority of immune cells from day 8 onwards were Iba^+^ microglia negative for CXCR4 [39]. As such, the mode of action of AMD3100 probably involves two levels of interaction. First, it modulates the inflammatory response in the acute phase which in turn affects the primary accumulation of immune cells. Subsequently, it regulates mechanisms of neuronal plasticity remodeling in the recovery phase through its interaction with CXCR4^+^ cells. Such cells could potentially be neurons, microglia, or astrocytes of the glial scar that constitutively or transiently express CXCR4 [20].

However, it still remains to be determined how the chemokine receptor interacts mechanistically with GABA_A_ receptors. The fact that we observed no difference in transcript amounts of GABA_A_ receptor subunits between sham/naïve and PT animals 14 days after stroke may indicate a stabilization of the inhibitory tonus in the peri-infarct area which partially is mediated by CXCR4. Similar transcript levels of CXCR4 observed both 2 weeks after PT with and without AMD3100 treatment might correspond to a stable CXCR4 expression in neurons. In addition, we found no changes in transcriptional activity of GABA_A_ subunit genes in the lesioned ipsilateral hemisphere, ultimately retaining a stable GABA_A_ receptor assembly. Therefore, effects of AMD3100 on the expression of GABA_A_ receptor subunits most likely seem an indirect effect possible through regulation of the fractalkine/CX3CR1 pathway [43].

Differential patterns of the CXCR4 expression on GABAergic interneurons [40, 41] in the ipsilateral and contralateral hemisphere may also have an effect on GAD67 levels. In vitro studies have demonstrated that activation of the CXCR4 signaling through CXCL12 induces the expression of GAD67 in embryonic hippocampal neurons [44]. In line with these data, we found that treatment with AMD3100 resulted in a decrease of GAD67 levels in the peri-infarct area. Moreover, treatment with AMD3100 could possible prevent the establishment of the GABAergic phenotype in migrating neural precursor cells or residing viable neurons in the peri-infarct area, ultimately suppressing the formation of GABAergic interneurons [40, 45]. This effect was not found in the contralateral cortex, possibly due to a different mechanisms of action between healthy tissue and tissue adjacent to the infarct core.

An endogenous down-regulation of GAD67 in the contralateral cortex after PT might be achieved as a result of the interhemispheric imbalance [36]. Increased neuronal activity is observed in the homotopic motor regions of the contralateral hemisphere, reflected by reduced GAD67 levels. Attenuated inhibition due to treatment with AMD3100 elevates neuronal activity in the lesioned hemisphere and thereby might contribute to re-establishes an equilibrium between the two hemispheres. This process includes the normalization of GAD67 levels and the regulation of the β3 subunit in the contralateral hemisphere. In addition, up-regulation of GAD65 in the contralateral cortex by treatment with AMD3100 indicates an increased inhibitory tonus.

### Cross-Talk Between the CX3CR1 Pathway and the GABAergic System

Several studies have focused on the involvement of the CX3CL1/CX3CR1 pathway in stroke pathophysiology, presenting ambiguous conclusions on whether activation of the chemokine cascade possesses a neuroprotective or detrimental role [22, 23, 25]. Our results show that CX3CR1 deficiency alters the expression of GABA_A_ subunits in the ipsilateral peri-infarct area and contralateral cortex. In addition, GABA_A_ receptor regulation by the CX3CR1 pathway may include post-translational modifications which may affect membrane stability, assembly dynamics, and degradation rate [37–39]. Interestingly, activation of the pathway in cells of the rat midbrain raphe nuclei increased inhibitory postsynaptic currents (IPSC) in serotoninergic neurons [46]. Susceptibility to inhibitory currents might have been related to an increase in the number of post-synaptic receptors, a theory that could explain the alterations of GABA_A_ subunits seen in our study. Under physiological conditions, the absence of CX3CR1 may promote a compensatory increase of GABA_A_ receptors preserving a sufficient inhibitory tonus in the contralateral hemisphere.

Moreover, administration of CX3CL1 to cortical tissue derived from patients with mesial temporal lobe epilepsy, a pathological condition associated with impaired GABA_A_ inhibitory signaling, attenuated use-dependent decrease (rundown) of GABA-evoked currents suggesting GABA_A_ receptor desensitization or subunit alterations by the CX3CR1 chemokine pathway [47]. It should also not been neglected that alterations of GABA-currents and synaptic modifications might have been induced by microglia [48]. Therefore, further studies are required to elucidate the role of microglia in the regulation of GABA_A_ receptor subunits in *cx3cr1* knockout mice and respective wild-type littermates.

## Conclusion

Our study shows that treatment with the CXCR4 antagonist AMD3100 modulates the expression of GABA_A_ receptor subunits and regulates the level of GAD65 and 67 in the post-ischemic brain. In addition, we also found that mice deficient for CX3CR1 have altered expression levels of GABA_A_ receptor subunits. Together, results indicate that the CXCR4/CX3CR1 pathways interact with components of GABAergic neurotransmission enhancing mechanisms of neuronal plasticity required to regain lost neurological function.

## Supplementary Information

Below is the link to the electronic supplementary material.

Supplementary file1 (PDF 1367 KB)

## Data Availability

All the datasets and materials supporting the conclusions of this article are presented in the manuscript and its online resource information file.

## Abbreviations

AMD3100: 1,1′-[1,4-Phenylenebis(methylene)]bis[1,4,8,11-tetraazacyclotetradecane]
CX3CL1: C-X3-C Chemokine ligand 1
CX3CR1: C-X3-C chemokine receptor type 1
CXCL12: C-X-C Chemokine ligand 12
CXCR4: C-X-C chemokine receptor type 4
CXCR7: C-X-C chemokine receptor type 7
E/I: Excitatory/inhibitory
GABA: γ-Aminobutyric acid
GAD67: Glutamate decarboxylase 67
GAD65: Glutamate decarboxylase 65
IPSC: Inhibitory postsynaptic currents
PT: Photothrombosis
tMCAO: Transient middle cerebral artery occlusion
