# Supplementary material for: Effect of Anti-inflammatory Treatment with AMD3100 and CX3CR1 Deficiency on GABAA Receptor Subunit and Expression of Glutamate Decarboxylase Isoforms After Stroke
Source: Mol Neurobiol. 2021 Aug 20;58(11):5876–89. doi: 10.1007/s12035-021-02510-x (PMC8599239; doi:10.1007/s12035-021-02510-x)
Supplement: Supplementary file 1 — Supplementary file1 (PDF 1367 KB) [file 12035_2021_2510_MOESM1_ESM.pdf]

Effect of anti-inflammatory treatment with AMD3100 and CX_3CR1 deficiency on $GABA_A$ receptor subunit and GAD isoforms expression after stroke

Georgios Michalettos[1], Helene L Walter[1,3], Ana Rita Pombo Antunes[1], Tadeusz Wieloch[1], Daniela Talhada[1] and Karsten Ruscher[1,2]

[1]Laboratory for Experimental Brain Research, Division of Neurosurgery, Department of Clinical Sciences, Wallenberg Neuroscience Center, Lund University, BMC A13, 22184, Lund, Sweden

[2]LUBIN Lab - Lunds Laboratorium för Neurokirurgisk Hjärnskadeforskning, Division of Neurosurgery, Department of Clinical Sciences, Lund University, Sweden

[3]Department of Neurology, Faculty of Medicine and University Hospital Cologne, University of Cologne, Cologne, Germany.

MOLECULAR NEUROBIOLOGY

SUPPLEMENTARY MATERIAL

*Corresponding Author: Dr. Karsten Ruscher, Associate Professor, Laboratory for Experimental Brain Research, Wallenberg Neuroscience Center, Lund University, BMC A13, S-22184 Lund, Sweden. Tel +46-46-2220613. email: karsten.ruscher@med.lu.se

Table 1 Sequences of gene specific primers and associated amplicon lengths

Gene	Forward (5´→3´)	Reverse (5´→3´)	Product size (bp)
**GAPDH**	TTCTCAAGCTCATTTCC TGGTATG	GGATAGGGCCTCTCTT GCTCA	143
**GABA(A) α3**	GTTCTCACCATGACCAC CTT	GTTGGAGCTGCTGGTG TTT	153
**GABA(A) δ**	CTCCCTGACACCTTCAT CGT	GTACTTGGCGAGGTCC ATGT	153
**GABA(A) β3**	GCCTTGATGACAGCCC TTTA	CATTGGCAACACAACC ACTC	174
**GAD67**	GAACAACCATGGTGGG CTAC	GCCGATGATTCTGGTTC TGT	166
**CXCR4**	TCAGTGGCTGACCTCCT CTT	CTTTTCAGCCAGCAGTT TCC	222

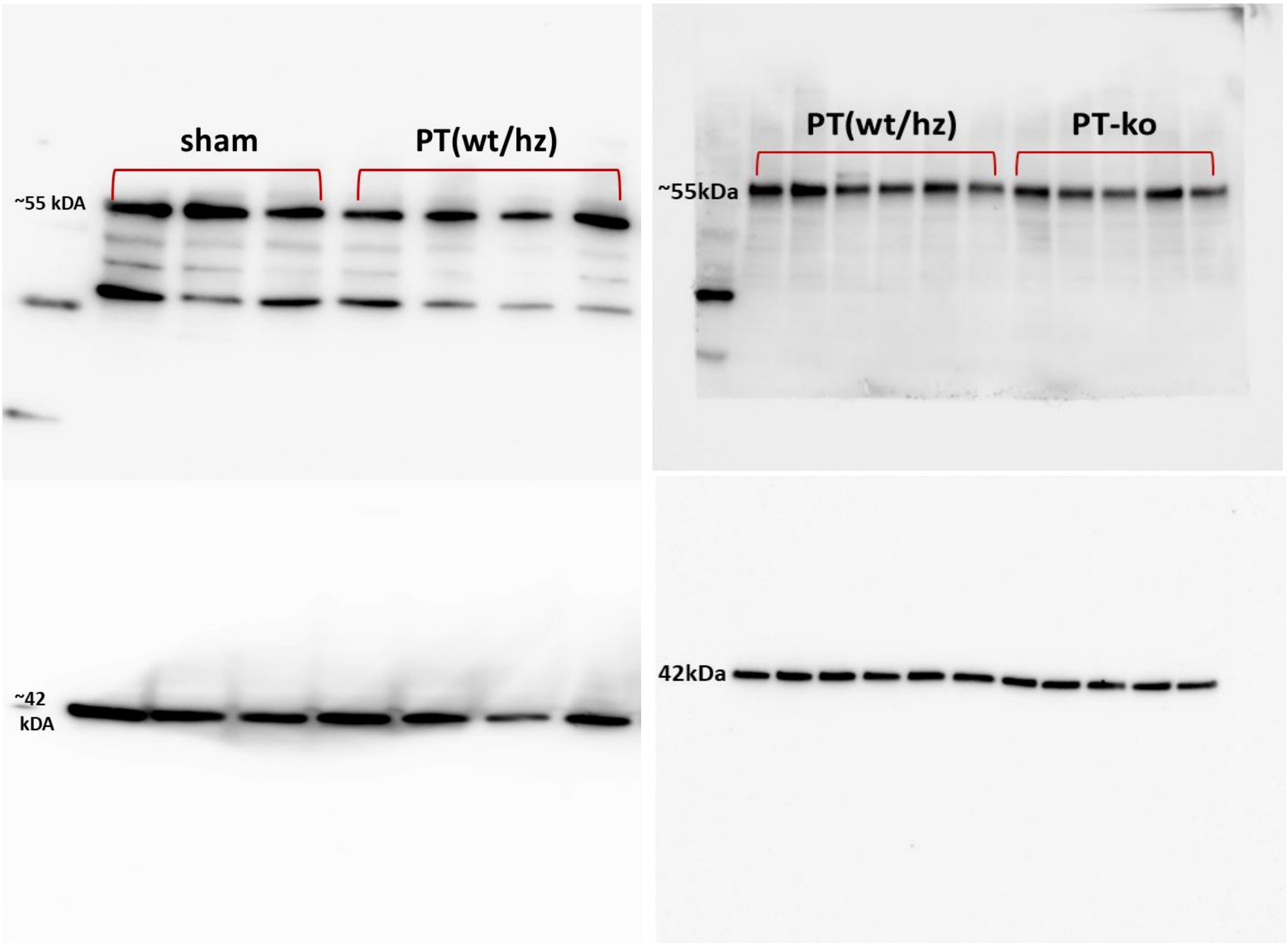

Supp. Figure 1 Western Blot of the $GABA_A$ α3 subunit in the contralateral cortex of mice which underwent sham surgery and stroke-mice of day 14 after PT regarding CX3CR1 deficiency. Corresponding band size were 55kDa for α3 subunit and 42kDA for β-actin.

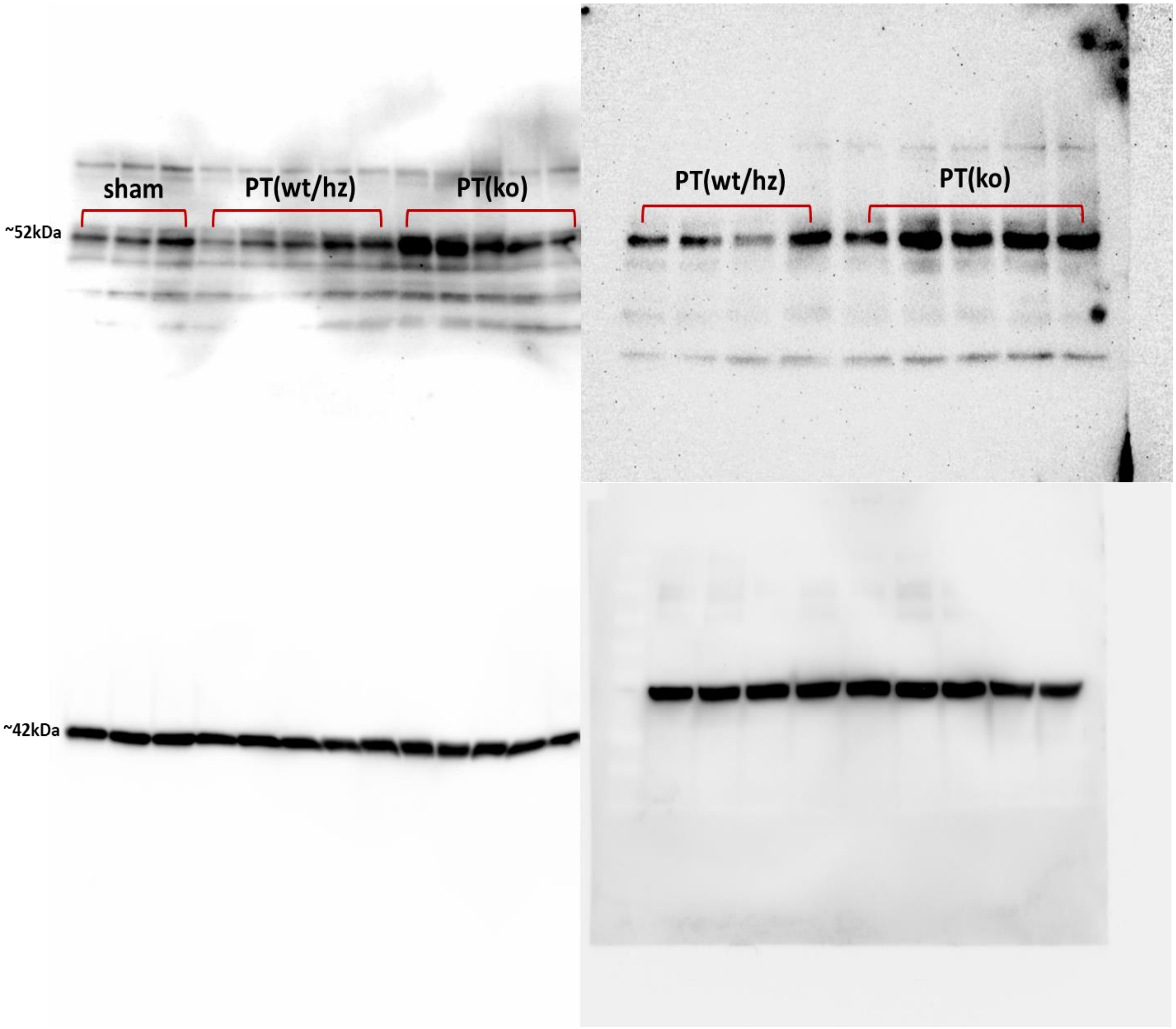

Supp. Figure 2 Western Blot of the GABA$_A$ δ subunit in the contralateral cortex of mice which underwent sham surgery and stroke-mice of day 14 after PT regarding CX3CR1 deficiency. Corresponding band sizes were approximately 52kDa for δ subunit and 42kDA for β-actin. The comparison PT (wt/hz) VS PT (ko) was repeated in a second Western Blot in order to confirm results.

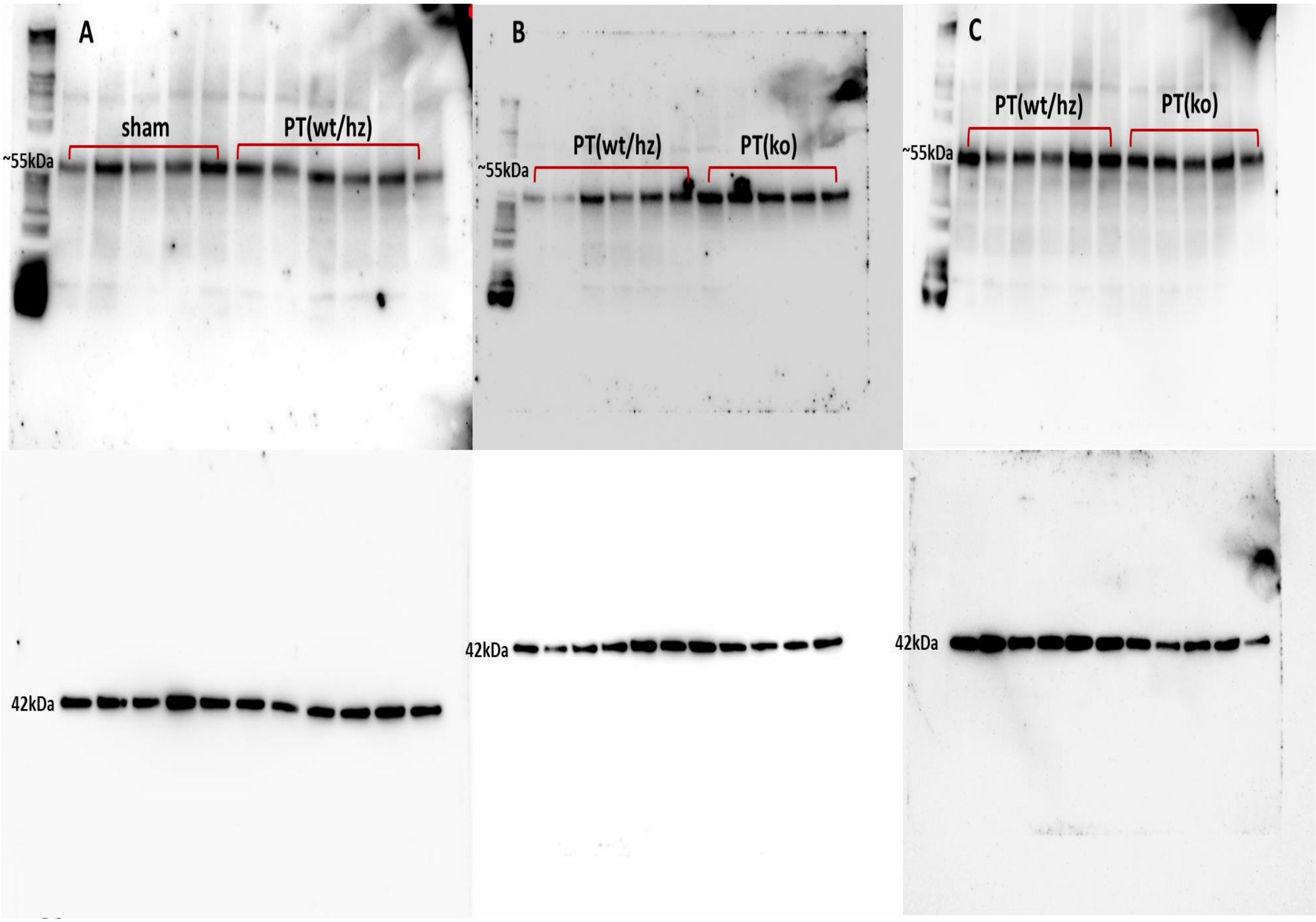

Supp. Figure 3 Western Blot of the $GABA_A$ β3 subunit in the contralateral cortex of mice which underwent sham surgery and stroke-mice of day 14 after PT regarding CX3CR1 deficiency. **(A)** Comparison between contralateral cortex of sham and stroke mice and **(B, C)** comparison between wt/hz littermates and ko mice which underwent stroke. Corresponding band sizes were approximately 55kDa for β3 subunit and 42kDA for β-actin. The comparison PT (wt/hz) VS PT (ko) was repeated twice **(B, C)** in order to confirm results.

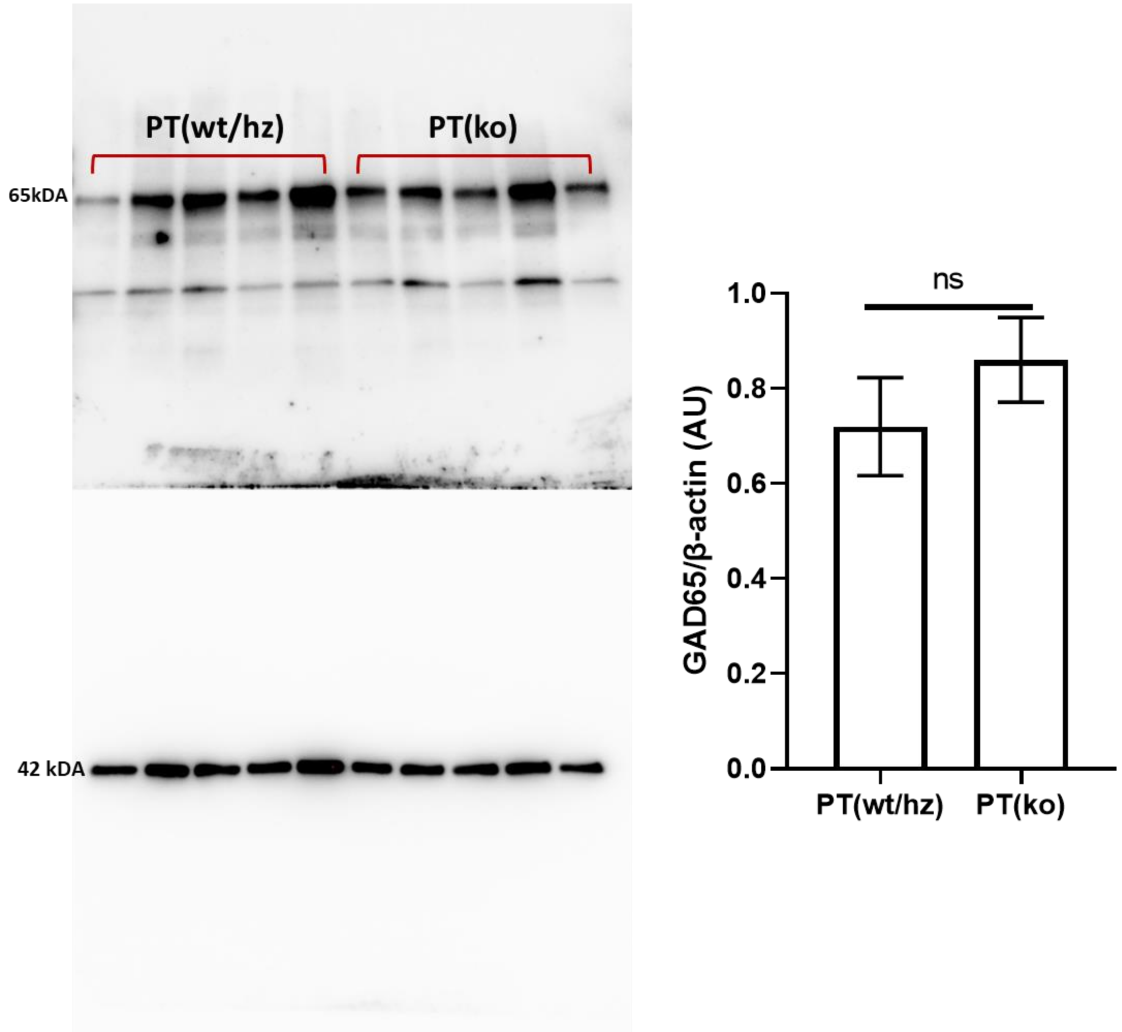

Supp. Figure 4 Western Blot of GAD65 in stroke-mice of day 14 after PT in the contralateral cortex regarding the effect of CX3CR1 deficiency. Comparison between wt/hz and ko stroke mice. Corresponding band size were 65kDa for GAD65 and 42kDA for β-actin. No significant differences were observed with Student's t test (*p = 0.33*).

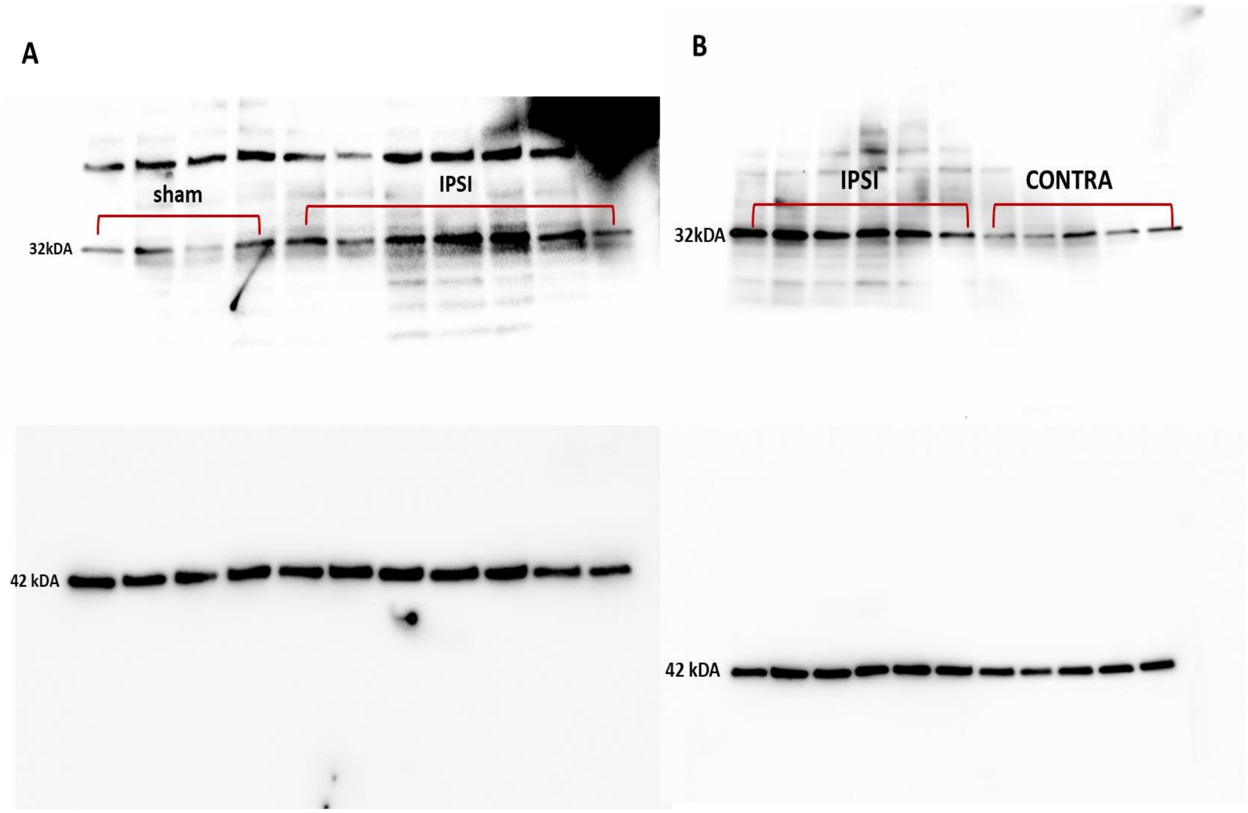

Supp. Figure 5 Western Blot of CXCR4 in stroke-mice of day 14 after PT regarding the effect of AMD3100 treatment. **(A)** Comparison between ipsilateral cortex of sham and stroke mice and **(B)** comparison of ipsilateral vs contralateral cortex of stroke mice. Corresponding band size were 32kDa for CXCR4 and 42kDA for β-actin.

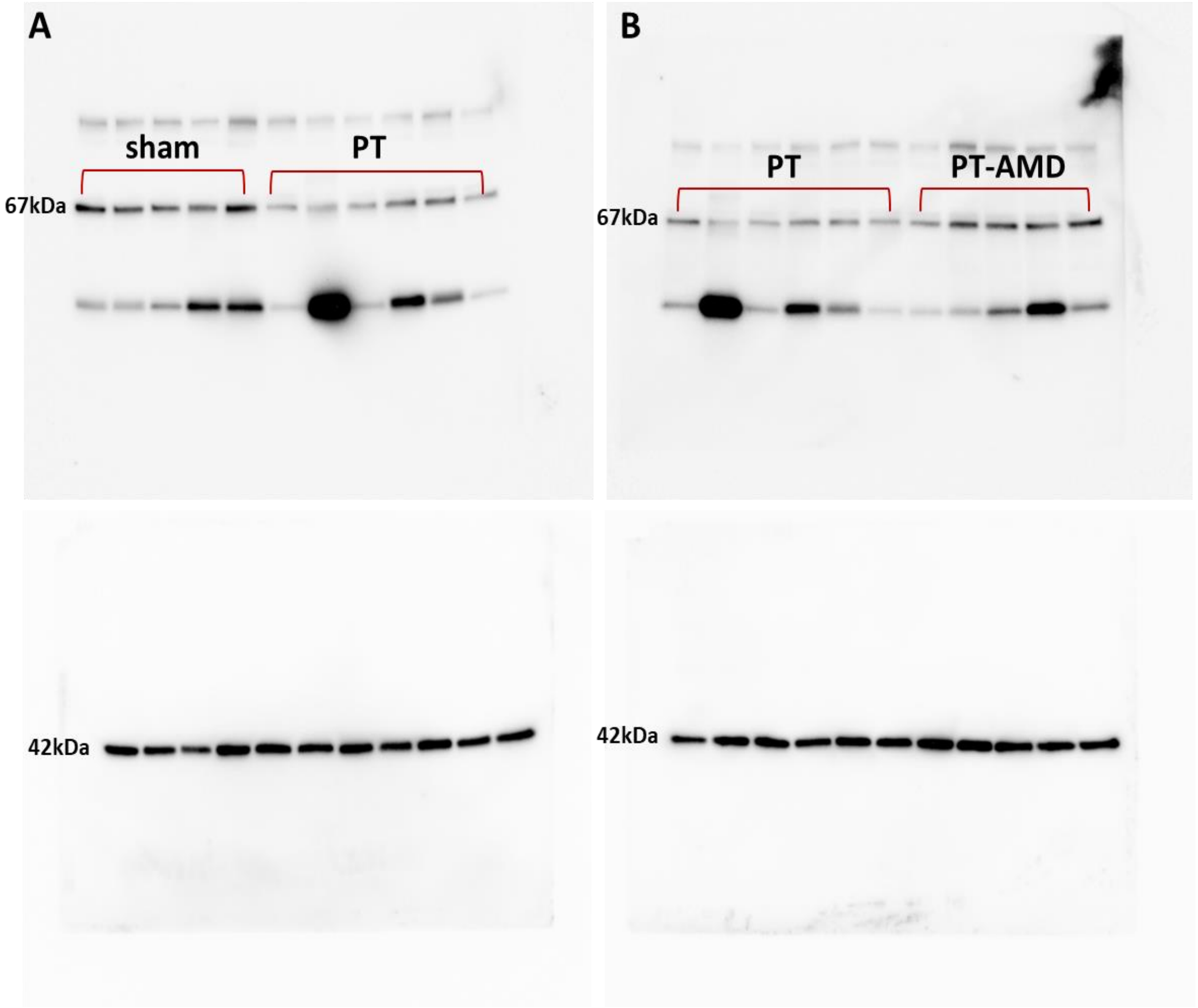

Supp. Figure 6 Western Blot of GAD67 in the **contralateral** cortex of stroke-mice of day 14 after PT regarding the effect of AMD3100 treatment. **(A)** Comparison of sham vs PT and **(B)** comparison of stroke-mice treated with saline or AMD3100. Corresponding band size were 67kDa for GAD67 and 42kDA for β-actin.

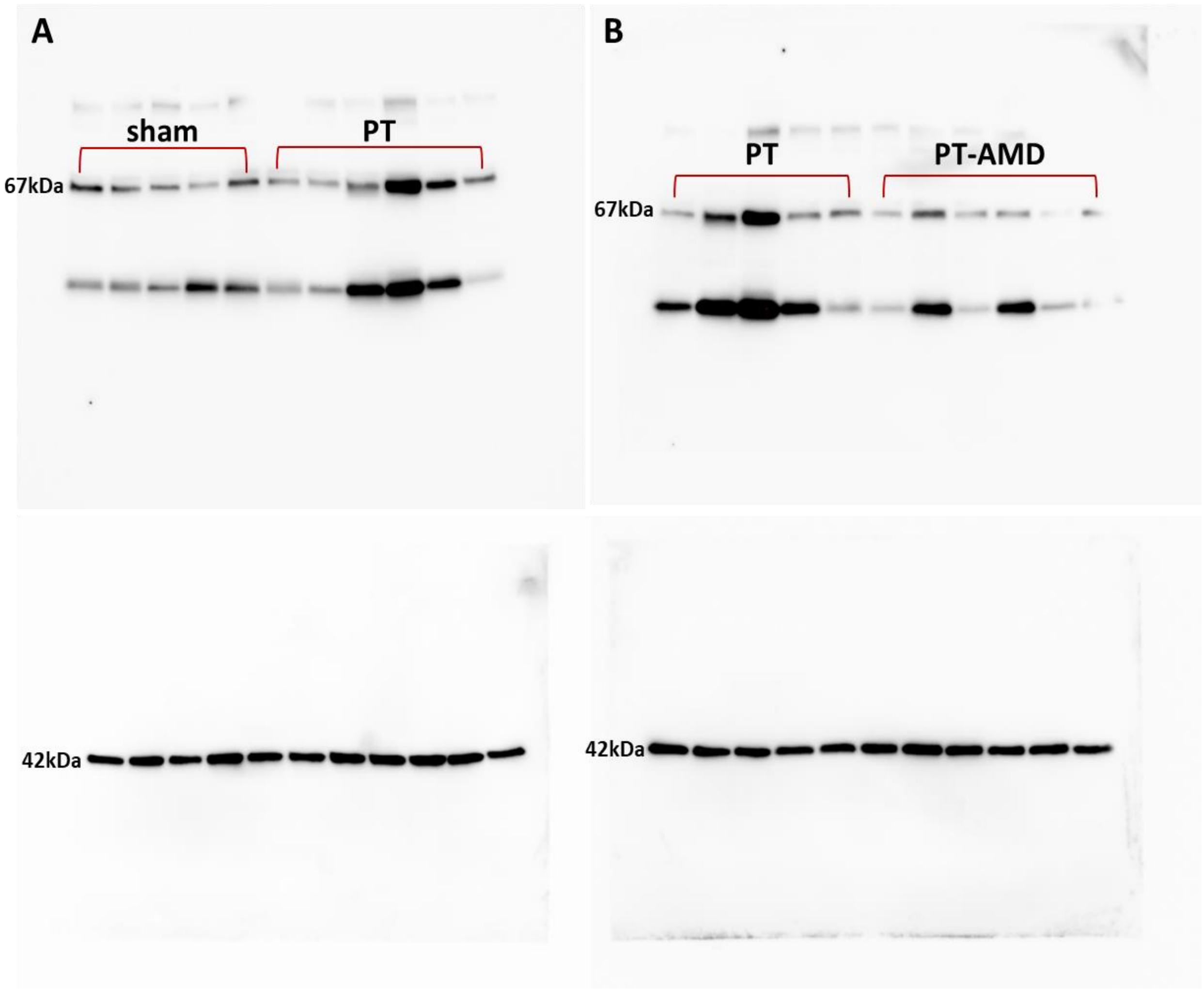

Supp. Figure 7 Western Blot of GAD67 in the **ipsilateral** cortex of stroke-mice of day 14 after PT regarding the effect of AMD3100 treatment. **(A)** Comparison of sham vs PT and **(B)** comparison of stroke-mice treated with saline or AMD3100. Corresponding band size were 67kDa for GAD67 and 42kDA for β-actin.

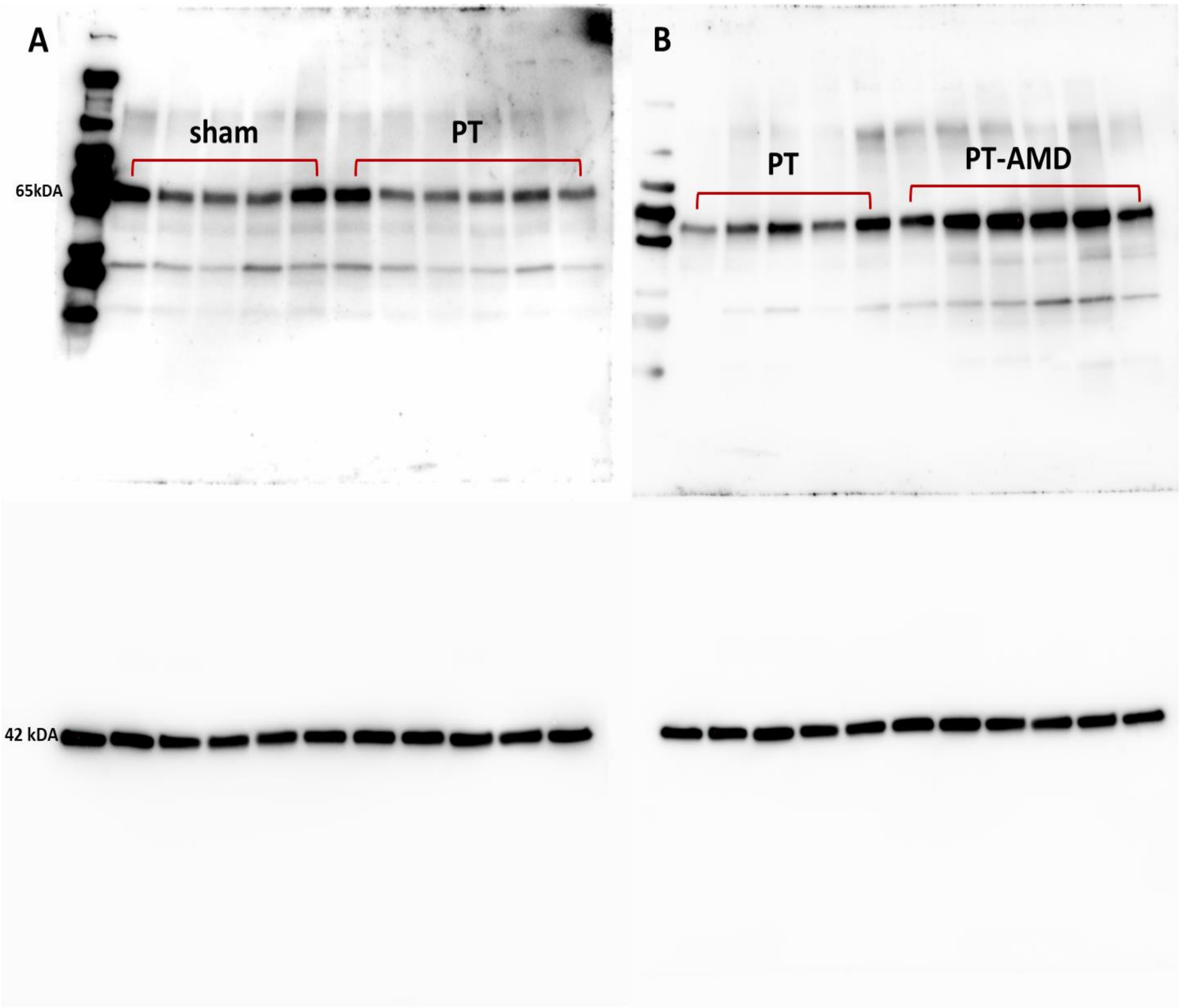

Supp. Figure 8 Western Blot of GAD65 in the **contralateral** cortex of stroke-mice of day 14 after PT regarding the effect of AMD3100 treatment. **(A)** Comparison of sham vs PT and **(B)** comparison of stroke-mice treated with saline or AMD3100. Corresponding band size were 65kDa for GAD65 and 42kDA for β-actin.

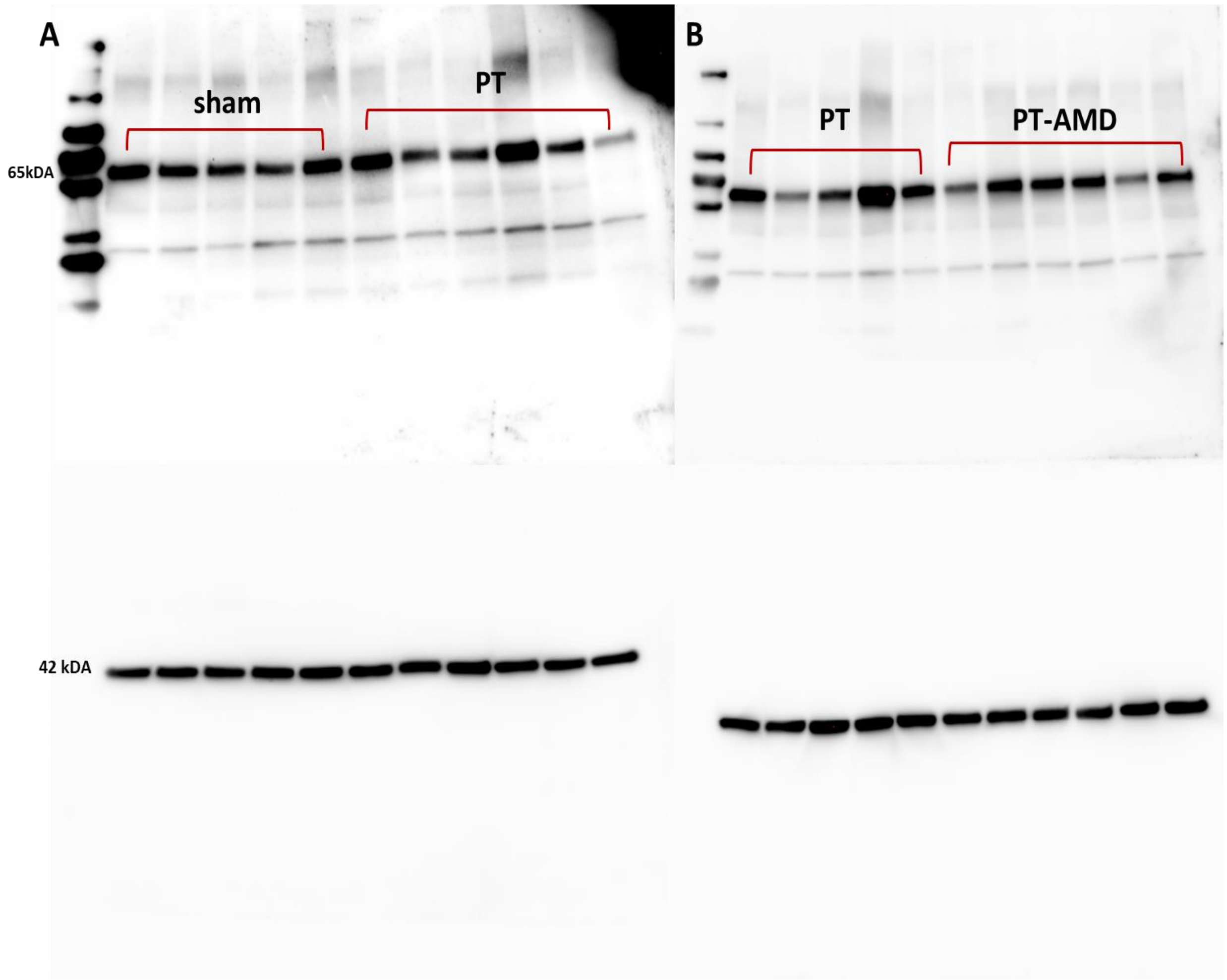

Supp. Figure 9 Western Blot of GAD65 in the **ipsilateral** cortex of stroke-mice of day 14 after PT regarding the effect of AMD3100 treatment. **(A)** Comparison of sham vs PT and **(B)** comparison of stroke-mice treated with saline or AMD3100. Corresponding band size were 65kDa for GAD65 and 42kDA for β-actin.

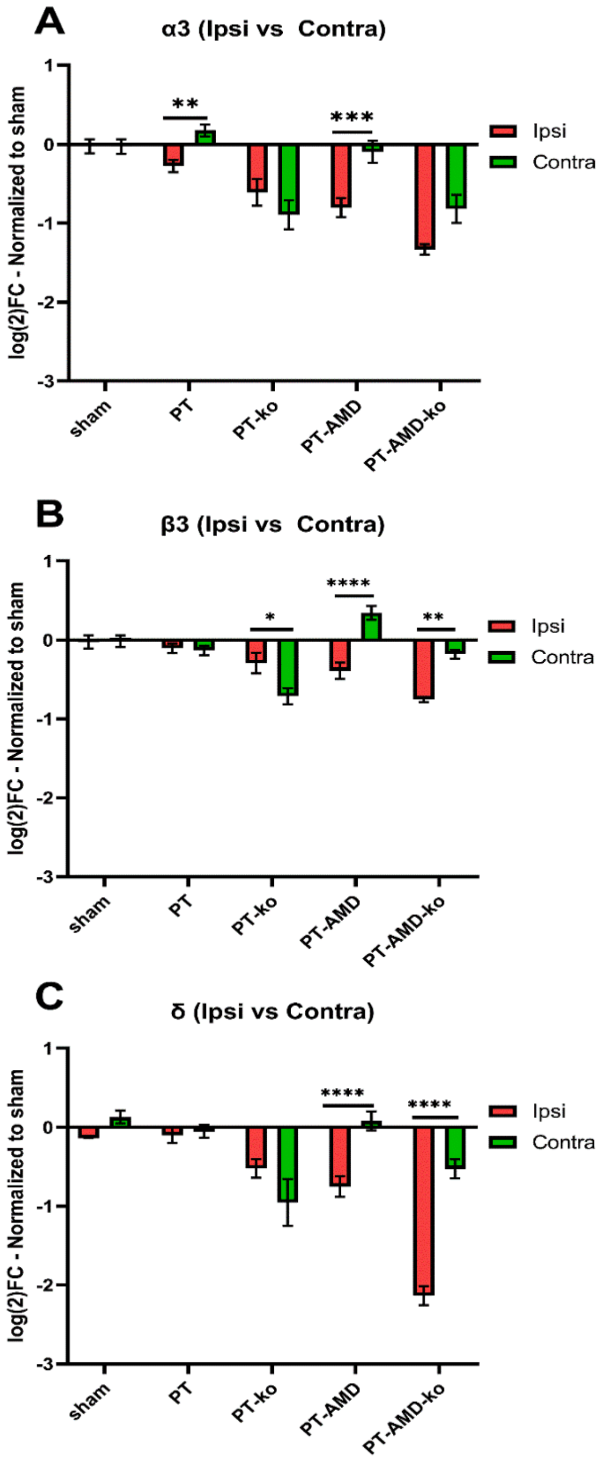

Supp. Figure 10. Comparison of mRNA levels between the two hemispheres under different experimental conditions mRNA levels of the α3 **(A)**, the δ **(B)** and the β3 **(C)** GABAA subunits in the contralateral and ipsilateral cortex of mice. All data depicted are normalized to expression levels of

sham/naive mice and shown as a log2 fold-change. Bars represent the mean ± S.E.M. Statistically significant differences were observed through Two-Way ANOVA and *post-hoc* Sidak's Multiple Comparisons (* $p < 0.05$, ** $p < 0.005$, **** $p < 0.0001$).
